# Identification and validation of cilia-associated molecular candidates deregulated in severe asthma

**DOI:** 10.1186/s12931-026-03548-y

**Published:** 2026-02-09

**Authors:** Maëva A Devilliers, Lynda Saber Cherif, Audrey Brisebarre, Ruby Chouquet, Ludivine Bralet, Julien Ancel, Alexandre Vivien, Emilie Luczka-Majérus, Arnaud Bonnomet, Nathalie Lalun, Camille Taillé, Xavier Dubernard, Jean-Claude Mérol, Christophe Ruaux, Myriam Polette, Gaëtan Deslée, Jeanne-Marie Perotin, Valérian Dormoy

**Affiliations:** 1 Université de Reims Champagne-Ardenne, INSERM, P3Cell UMR-S1250, Reims, France; 2 Université de Reims Champagne-Ardenne, INSERM, CHU de Reims, P3Cell UMR-S1250, Reims, France; 3 Université de Reims Champagne-Ardenne, INSERM, P3Cell UMR-S1250, URCATech, PICT, Reims, France; 4 Service de pneumologie et Centre de Référence des Maladies Pulmonaires rares, Hôpital Bichat, Groupe Hospitalier Universitaire AP-HP Nord, UMR 1149, Université Paris Cité, Paris, France; 5 Université de Reims Champagne-Ardenne, CHU de Reims, Reims, France; 6 Département d’Otorhinolaryngologie, Clinique Mutualiste La Sagesse, Rennes, France; 7 Institut universitaire de France (IUF), Paris, France

**Keywords:** Asthma, Airways, Cilia, Epithelial cells, NEK6, PHLDB2, SCNN1G

## Abstract

**Background:**

Asthma is characterised by a chronic inflammation and airway remodelling. The functionality of the asthma airway epithelium is altered, suggesting a central role in the pathophysiology. Cilia-associated abnormalities have been reported in the airway epithelium of asthmatic patients, but the mechanisms remain elusive. This study investigated cilia-associated dysregulations in cohorts of patients with severe asthma to identify and characterize key molecular drivers of epithelial airway remodelling.

**Methods:**

Transcriptomic data from epithelial bronchial brushing samples of three large cohorts of severe asthma patients and non-asthmatic were analysed: U-BIOPRED (GSE76226, *n* = 105), SARP (GSE63142, *n* = 81) and IMSA (GSE158752, *n* = 42). We focused on cilia-associated genes to highlight common differentially expressed genes in all three cohorts, comparing the non-asthmatic and severe asthma groups. Localisation and expression of the three most dysregulated genes were then validated on ex vivo and in vitro samples and correlated to epithelial airway remodelling features and clinical data.

**Results:**

Seventeen genes were significantly dysregulated between the non-asthma and severe asthma groups in all three datasets. We identified the three most dysregulated: PHLDB2 (12.7% [4.6–22.4], *p* < 0.05), NEK6 (8.4% [5.5–12.8], *p* < 0.05) and SCNN1G (6.8% [4.3–9.9], *p* < 0.05) as molecular candidates. In ex vivo severe asthma bronchial biopsies, the protein levels were upregulated 1.2-fold for PHLDB2 (*p* = 0.2844), 2.4-fold (*p* < 0.01) for NEK6, and 2.0-fold for SCNN1G (*p* < 0.05). The expressions of these candidates were correlated with epithelial remodelling features in both groups. In vitro, the expression of SCNN1G was upregulated in airway epithelial cells upon IL-13 stimulation, suggesting that SCNN1G may be dynamically regulated during epithelial differentiation under pro-inflammatory conditions. Finally, protein expression of PHLDB2, NEK6 and SCNN1G in fully differentiated air-liquid interface culture was associated with epithelial remodelling features with or without inflammatory stimulation.

**Conclusion:**

This study highlights the dysregulation of three cilia-associated genes, PHLDB2, NEK6, and SCNN1G, in the bronchial epithelium of patients with SA. Their association with epithelial remodelling features and altered differentiation under inflammatory conditions highlights their potential involvement in asthma pathogenesis and their potential as predictive biomarkers or therapeutic targets in severe asthma.

**Supplementary Information:**

The online version contains supplementary material available at 10.1186/s12931-026-03548-y.

## Introduction

 Asthma is a chronic inflammatory disorder of the airways with a prevalence of 3,340 cases per 100,000 people worldwide [1]. It is typically characterised by common symptoms such as wheezing and shortness of breath, which are associated with airway remodelling and hyperresponsiveness [2]. Asthma is a heterogeneous disease in terms of phenotypes and endotypes. To date, asthmatics are largely classified based on the inflammatory context, allowing for long-term control medications considering the trigger, age, and symptoms [3, 4]. Nonetheless, uncontrolled asthma or severe asthma affects almost 10% of the asthma population and has a significant impact on morbidity. It is characterised by frequent exacerbations, treatment resistance and impaired lung function [5].

The functionality of the airway epithelium is altered in asthma. The dysfunction of the mucociliary clearance suggests a central role of the airway epithelial remodelling in asthma physiopathology [6]. If the identification of cilia is standardised with the detection of ubiquitous intraciliary proteins such as the small GTPase ARL13B or the highly conserved microtubule stabilisation program involving acetylation, very sparse studies have evidenced possible modifications on these biomarkers (except in ciliopathies) and not in the respiratory context [7, 8]. Nonetheless, cilia abnormalities have been reported in the bronchial epithelium of asthmatic patients, including a decrease in ciliary beat frequency and ultrastructural abnormalities in association with disease severity [9]. The underlying mechanisms remain unclear. In contrast, mucociliary clearance defects were associated with a T2-high inflammatory phenotype [10]. The stimulation of primary tracheal epithelial cells with IL-13 enhanced cellular remodelling of the mucociliary epithelium and altered ciliated cell differentiation [11]. Furthermore, experimental studies have demonstrated the repression of the cilia-associated genes multicilin (MCIDAS) and Forkhead Box J1 (FOXJ1) in the presence of IL-13, inducing a severe depletion of ciliated cells in a normal human bronchial epithelial in vitro model [12]. Additionally, the destabilisation of FOXJ1 mRNA induces the indirect development of an asthma-like phenotype in a mouse model [13].

In a physiological context, we have previously demonstrated that primary cilia are required for the differentiation of multiciliated cells in the respiratory epithelium and that inhibition of primary cilia leads to remodelling features [14, 15]. In COPD patients, we observed an increase in primary ciliated airway epithelial cells (AEC) in remodelled bronchial epithelia, and we defined a ciliopathy-associated COPD endotype [16, 17]. More recently in severe asthma, we have identified a novel endotype centred on the dysregulation of the cilia-associated gene signature that may impact epithelial remodelling [18]. Altogether, these findings suggest a tight link between cilia-associated defects and epithelial remodelling in severe asthma airways. Therefore, we investigate here the cilia-associated transcriptomic dysregulations in large cohorts of patients with severe asthma to identify potential key molecular drivers involved in the pathological process underlying bronchial epithelial remodelling.

## Materials and methods

### Gene selection

Human cilia-associated genes (*n* = 879) were extracted from UniProtKB (release 2022_03) considering all reviewed human « Cilia », « Ciliopathy », and « Basal bodies » proteins in the database (as described in a previous study [18]).

### RNAseq analyses

Previously published publicly available datasets of gene expression of epithelial bronchial brushings obtained from healthy control (HC) and severe asthmatic (SA) patients were collected (GEO database; accession numbers: GSE76226, GSE63142, GSE158752). HC and SA patients were included as described in the original publications, respectively *n* = 44 vs. *n* = 61 in GSE76226, *n* = 27 vs. *n* = 56 in GSE63142, *n* = 17 vs. *n* = 25 in GSE158752 [19–22]. In each cohort, the expression levels of cilia-associated genes were analysed between HC and SA groups. The fold change (SA/HC) was calculated using the mean expression values of each group, and genes with an FDR-adjusted p-value < 0.05 were considered significantly differentially expressed (DEGs). The differences between the two groups were determined using Student’s t-test and Benjamini-Hochberg FDR correction and expressed as fold-change SA/HC.

### Deconvolution

A multi-subject single-cell (MuSiC) deconvolution of the bulk RNA-seq data from the three cohorts GSE76226, GSE63142 and GSE158752 was performed using MuSiC (version 1.0.0) implemented in R (version 4.4.2). The reference dataset for deconvolution was a publicly available single-cell RNA-seq dataset from the Human Lung Cell Atlas (https://explore.data.humancellatlas.org/projects/c0518445-3b3b-49c6-b8fc-c41daa4eacba) in the context of asthma, including 15 major cell types [23]. Analyses were conducted separately for the HC and SA groups within each bulk RNA-seq cohort. The following R packages were used: *BiocManager* (version 1.30.26), *Biobase* (version 2.66.0), *TOAST* (version 1.20.0), *SingleCellExperiment* (version 1.28.1), *SummarizedExperiment* (version 1.36.0), *devtools* (version 2.4.6), *zellkonverter* (version 1.16.0) and *reticulate* (version 1.44.0). For each bulk RNA-seq dataset, deconvolution was performed using only the genes shared with the single-cell reference dataset, corresponding to 16,226 genes for GSE76226, 16,931 for GSE63142, and 20,018 for GSE158752.

### Human subjects

Non-asthmatic (NA) patients scheduled for bronchial biopsies were prospectively recruited in accordance with standards established and approved by the institutional review board of the University Hospital of Reims, France (IRB Reims-CHU 20110612), and were included in the cohort for research and innovation in chronic inflammatory respiratory disease (RINNOPARI, NCT02924818). Severe asthma (SA) patients were recruited in the Respiratory Diseases Department of the Bichat Hospital (Paris, France). Bronchial biopsies were performed in the lower right lung lobe before bronchial thermoplasty [24]. This protocol (ASMATHERM, NCT01777360) was approved by the local institutional review board (No. 2012-Sept-13003). Patients with other respiratory diseases (COPD, cystic fibrosis, bronchiectasis or pulmonary fibrosis) and recent exacerbations (< 6 weeks) were excluded. For all patients, age, gender, smoking history, comorbidities, pulmonary function test results and treatments were recorded at inclusion.

Human primary airway epithelial cells (AEC) were isolated from nasal polyps of non-asthmatic (*n* = 6) and asthmatic patients (*n* = 6) for air-liquid interface (ALI) cultures. Nasal polyps were resected at the University Hospital of Reims and the Clinic *La Sagesse* of Rennes, and included in the Biological Collection (DC-2012–1583, French Ministry of Research), approval n°21–775 of the Institutional Review Board 00003888 Inserm. All subjects gave their written informed consent.

### Human primary airway epithelial cell cultures

AEC were dissociated by overnight pronase incubation at 0.5 mg/mL (P5147, Sigma-Aldrich) and counted with ADAM (NanoEnTek) according to the manufacturer’s instructions. One million cells were seeded on a 10 cm Petri dish until cell confluency. After one passage, 50,000 cells were seeded on 12-well plates containing 0.4 μm Transwells (3460, Corning, Fisher Scientific) coated with 0.2 mg/ml collagen type IV from human placenta (C7521, Sigma-Aldrich) to establish ALI cultures.

CnT-17 media (CnT17, CellnTec) was used for initial proliferation in apical and basal chambers. Upon reaching cell confluence, the apical medium was removed, and PneumaCult-ALI (PnC-ALI, 05001, StemCell) medium was used in the basal chamber. The culture medium was changed three times a week, and cells were kept for 14 and 28 days in incubators at 37 °C, 5% CO_2_. There was no difference in the proliferative index of asthmatic and non-asthmatic cultures. IL-13 chronic treatment at 3 ng/mL (I1771, Sigma-Aldrich, diluted in sterile water) was used as an inflammatory stimulus. IL-13 was added to the culture basal medium with every medium change from 2 days of ALI culture until arrest. After 14 or 28 days of ALI culture, cells were fixed in formalin 4% (4087736, Microm Microtech, pH 7) and embedded in paraffin.

### Transepithelial electrical resistance (TEER) measurements

Transepithelial electrical resistance (TEER) was evaluated at ALI-14 and ALI-28 using an EVOM2 resistance meter with an STX2 electrode (World Precision Instruments Hitchin) at room temperature. The electrode was equilibrated in PnC-ALI for 2 h at room temperature before measurement. One mL of PnC-ALI was added to the apical compartment, and triplicate measurements were performed per sample. Data were corrected for blank values and area. Average resistance was subtracted from the measured value of every well according to data acquired on cell-free permeable supports, and results are presented as resistance per surface (Ω/cm²).

### Immunofluorescent stainings

Immunofluorescent stainings were performed on formalin-fixed paraffin-embedded (FFPE) bronchial biopsies and culture inserts. Three µm sections were processed for these immunostainings. FFPE section slides were deparaffinised, and antigen retrieval was performed in 1% citrate buffer (pH 6.0; *Vector Antigen unmasking solution*, H-3300, Vector Laboratories) for 20 min at 120 °C. Then, the slides were blocked with 10% BSA in PBS for 1 h at room temperature. The sections were then incubated with the primary antibody (listed in Supplementary Material Table S1) overnight at 4 °C in 3% BSA in PBS. We also included negative control immunostaining for the 3 proteins of interest that we identified in this study (Supplementary Fig. S2). After washing with PBS, a second primary antibody was used for 1 h at room temperature. The sections were washed with PBS and incubated with the appropriate secondary antibodies in 3% BSA in PBS for 30 min at room temperature. NEK6 staining was amplified using biotin-conjugated secondary antibody (111-066-045, Jackson ImmunoResearch) with streptavidin Alexa Fluor 568 conjugate (S11226, Invitrogen). The DNA was stained with DAPI in PBS for 15 min at room temperature.

Micrographs were acquired by AxioImager Zeiss (20x Ph) with ZEN software (8.1, 2012) and processed with ImageJ (National Institutes of Health) and CellProfiler (version 4.2.8). For each patient, all available epithelium (up to 4 random fields) per section containing bronchi were taken. The biopsies contained a minimal epithelial abrasion and there was no difference between NA and SA. For each field, a threshold was established by subtracting the background and setting the minimum at 0. CC10, MUC5AC, NEK6, PHLDB2 and SCNN1G expressions were determined by the pixel mean grey values (pmgv) in the selected region of interest defined by the entire epithelium in the two groups (except for SCNN1G: the region of interest was defined by solely the basal cells of the epithelium). MUC5B-positive and p63-positive cells were counted in the epithelium and normalised to the total cell count using the “IdentifyPrimaryObjects” tool with “Otsu thresholding” from CellProfiler. The surface area of ciliated cells was determined by measuring ARL13B or acetylated tubulin expression on the apical surface of the epithelium, as described by us and others [15, 17, 25]. Epithelial height was assessed by measuring six fields of the epithelium.

### Statistics

Data were expressed as mean values +/- standard error of the mean (SEM) and percentages. Normality tests were assessed for each group using the Shapiro-Wilk test. Differences between the two groups were determined using the unpaired or paired Student’s test (two-tailed) for parametric data, the Mann-Whitney or Wilcoxon test for non-parametric data. The correlations were established with the Pearson test. A p-value < 0.05 was considered significant; * *p* < 0.05; ** *p* < 0.01; *** *p* < 0.001; **** *p* < 0.0001.

## Results

### Analysis of the cilia-related transcriptomic profile in severe asthma

**Fig. 1:**
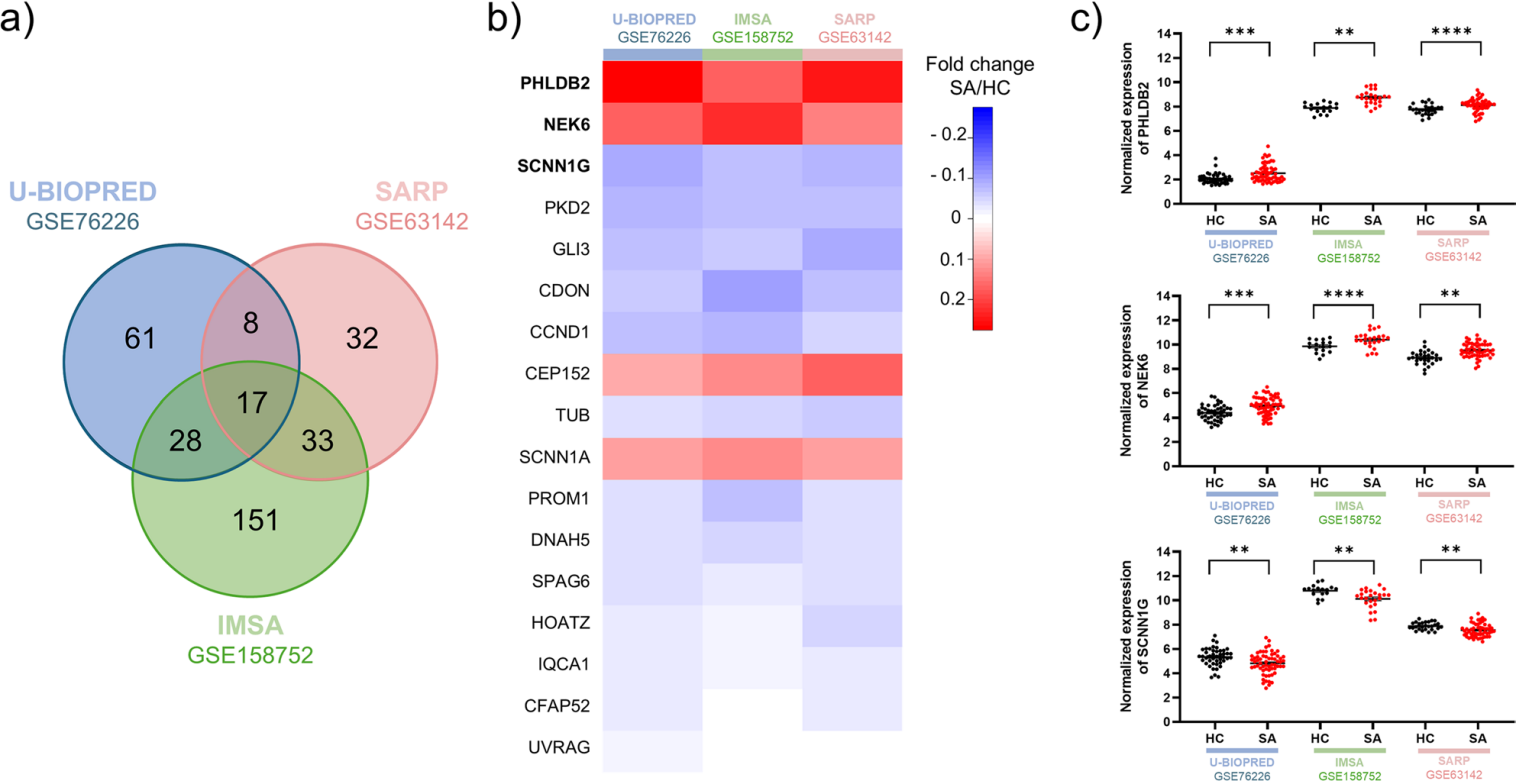
Analysis of cilia-associated bronchial brushing transcriptomes of SA patients. **a** Venn diagram showing the number of cilia-associated genes significantly dysregulated between HC and SA groups in all three datasets: GSE76226 (in blue), GSE63142 (in pink), and GSE158752 (in green). **b** Heatmap showing the fold changes SA/HC of the top 17 DEGs in the three datasets. Downregulated genes are in blue and upregulated genes are in red. DEGs are presented from most (top) to least (bottom) dysregulated. **c** Dot plots representing normalized expression of the three most DEGs, PHLDB2 (top panel), NEK6 (middle panel) and SCNN1G (bottom panel) between NA (HC, black) and SA (red) groups in all three cohorts. *p < 0.05; **p < 0.01; ***p < 0.001; ****p < 0.0001

Firstly, we performed a cross-expression analysis of the cilia-associated signature with 3 publicly available datasets obtained on airway epithelial (AEC) cells, including a total of 142 SA and 105 HC patients (respectively GSE76226, *n* = 61 vs. *n* = 44; GSE63142, *n* = 56 vs. *n* = 27; GSE158752, *n* = 25 vs. *n* = 34) [19–22]. Because the epithelial brushings could be heterogeneous, we performed a multi-subject single-cell (MuSiC) deconvolution of the bulk expressions with the labels of the Human Lung Consortium Atlas (HLCA) in the context of asthma, including 15 major cell types [23]. Over 75% of the deconvoluted transcriptomic prints matched AEC with similar proportions for the 3 datasets (Supplementary Fig. S1a). In particular, the collections were devoid of inflammatory or infiltrated cells such as dendritic cells, B cells, neutrophils, T and NK, or mast cells. Interestingly, approximately 50% of the deconvoluted transcriptomic prints matched ciliated cells, and there was no difference between HC and SA, nor between the 3 datasets (Supplementary Fig. S1b). Altogether, the biological collections appeared homogeneous with an enrichment of ciliated cells. 330 unique genes were found differentially expressed between HC and SA groups in at least one dataset (Fig. 1a). 114 cilia-associated genes were dysregulated in GSE76226, 229 in GSE63142 and 90 in GSE158752 databases, representing respectively 13.8%/10.8%/27.0% of all cilia-associated genes. 17 cilia-associated genes were significantly dysregulated between HC and SA groups in all three cohorts: four genes were upregulated (PHLDB2, NEK6, CEP152, SCNN1A) and thirteen were downregulated (SCNN1G, PKD2, CLI3, CDON, CCND1, TUB, PROM1, DNAH5, SPAG8, HOATZ, IQCA1, CFAP52, UVRAG) in SA patients (Fig. 1b). We then focused on the three most dysregulated genes across the three datasets: Pleckstrin homology-like domain family B member-2 (PHLDB2, 12.7% [4.6–22.4]), *p* < 0.05), NimA-related protein kinase 6 (NEK6, 8.4% [5.5–12.8], *p* < 0.05) and Epithelial sodium channel subunit gamma (SCNN1G, 6.8% [4.3–9.9], *p* < 0.05) (Fig. 1c).

### Localisation of PHLDB2, NEK6 and SCNN1G in bronchial epithelium

We explored the localisation and the association with histological and clinical features of the 3 candidates in bronchial biopsies obtained from NA (*n* = 5) and SA (*n* = 8) patients. Baseline characteristics, including age, sex, BMI, comorbidities, and smoking history, were similar with no significant differences observed between the NA and SA groups. The SA group was characterised by decreased FEV_1_/FVC ratio (0.5 ± 0.2 vs. 0.8 ± 0.1, *p* = 0.016), more frequent use of asthma inhaled treatments including CSI (8/8 vs. 0/5, *p* = 0.001), LABA (8/8 vs. 0/5, *p* = 0.001), LAMA (6/8 vs. 0/5, *p* = 0.021), and more frequent maintenance oral corticosteroids (5/8 vs. 0/5, *p* = 0.075) compared to the NA group (Table 1).

**Table 1:** Clinical characteristics of the patients included for FFPE immunostainings on bronchial biopsies

Clusters	SA	NA	*p* value
n	8	5	
*Demography and clinical characteristics*			
Age at inclusion (yrs)	49.6 ± 10.9	50.4 ± 14.8	0.915
Sex F/M	4/4	2/3	> 0.999
BMI^a^ (kg/m²)	29.6 ± 7.8	32.0 ± 6.8	0.587
Allergen sensitization	4 (50)	ND	
Total IgE^b^ (IU/mL)	324.1 [5-1300]	25.0 [13–37]	0.376
Blood eosinophils (cells/mm^3^)	321.8 ± 218.0	200.0 ± 70.7	0.258
Severe exacerbations* (last 12 months)	8 (100)	ND	
*Comorbidities*			
Allergic rhinitis	2 (25)	0 (0)	0.487
Nasal polyps	4 (50)	0 (0)	0.105
GERD^c^	3 (37.5)	1 (20)	> 0.999
*Smoking history*			
Never smokers	5 (62.5)	1 (20)	0.592
Current smokers	0 (0)	2 (40)	0.128
Former smokers	3 (37.5)	2 (40)	> 0.999
Pack-years	16.7 [10–20]	36.0 [20–50]	0.111
*Functional and biological characteristics*			
FEV_1_^d^ (%, predicted)	66.01 ± 17.5	82.3 ± 13.4	0.089
FEV_1_/FVC^e^	0.54 ± 0.16	0.77 ± 0.11	**0.016**
*Treatments*			
CSI^f^	8 (100)	0 (0)	**0.001**
LABA^g^	8 (100)	0 (0)	**0.001**
LAMA^h^	6 (75)	0 (0)	**0.021**
Maintenance oral corticosteroids	5 (62.5)	0 (0)	0.075
OCS^i^ daily dose	18.8 [15.0–20.0]	ND	
Biologics	1 (12.5)	0 (0)	> 0.999

^a^BMI: Body mass index; ^b^GERD: Gastroesophageal reflux disease; ^c^IgE: Immunoglobin-E; ^d^FEV1: Forced expiratory volume in one second; ^e^FVC: Forced vital capacity; ^f^CSI: Corticosteroids inhaled, ^g^LABA: Long-acting beta-2 agonists; ^h^LAMA: Long-acting muscarinic agonists; ^i^OCS: Oral corticosteroids. * Exacerbations that require systemic corticosteroids for > 3 days (or doubling dose if maintenance OCS). ND: Not determined

**Fig. 2:**
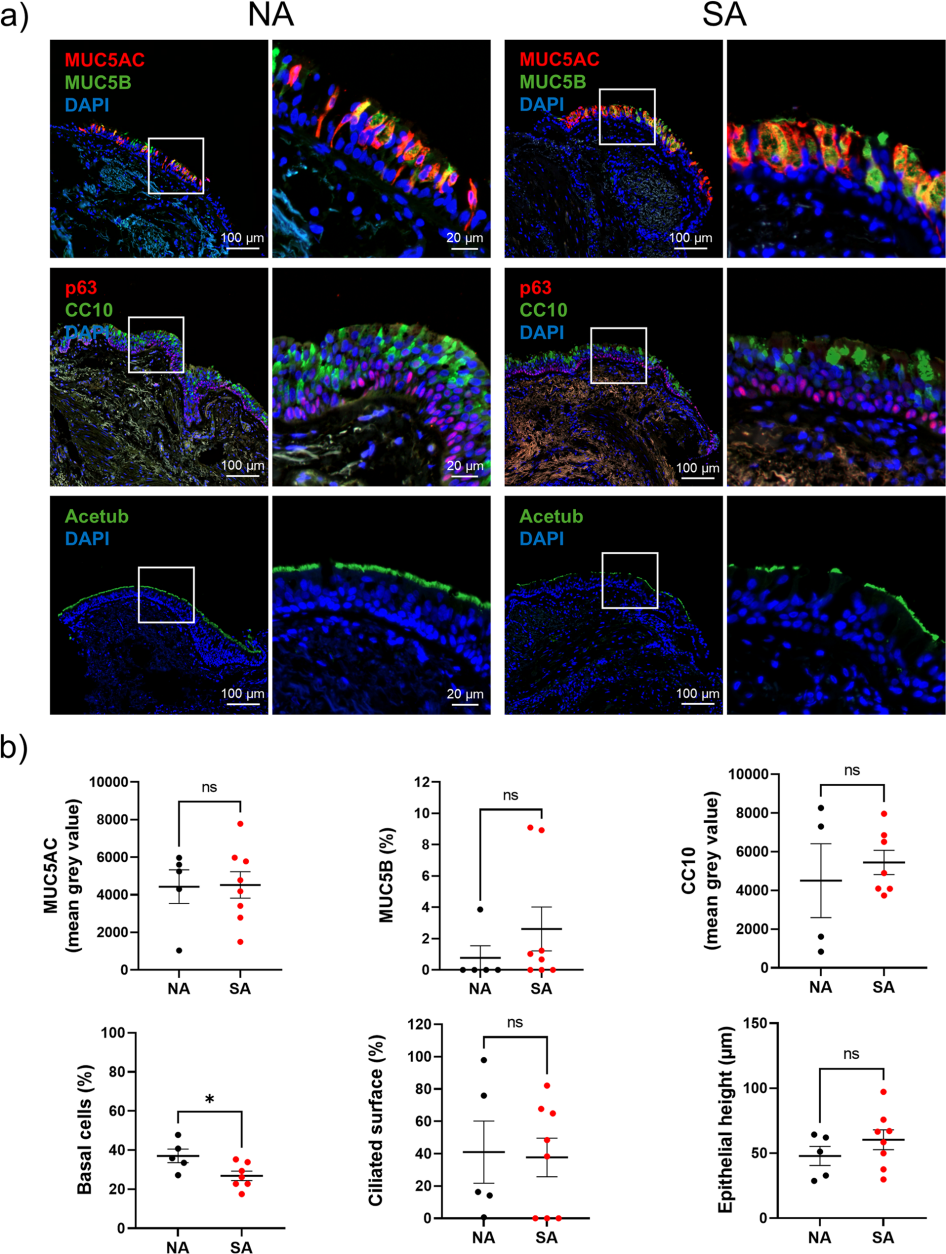
Analysis of bronchial remodelling between NA and SA. **a** Representative micrographs showing bronchial epithelia of non-asthmatic (NA) and severe asthmatic (SA) patients immunostained for epithelial remodelling features MUC5AC/MUC5B (top panel), p63/CC10 (middle panel), acetyltubulin (Acetub, bottom panel) and cell nuclei (DAPI, blue). Magnification corresponding to the selected area is shown. **b** Dot plots with mean ± SEM representing MUC5AC, MUC5B, CC10 expression, proportion of basal cells and ciliated surface, and epithelial height of NA (*n* = 5, black) and SA (*n* = 8, red) patients. **p < 0.01; ***p < 0.001; NA vs. SA

**Fig. 3:**
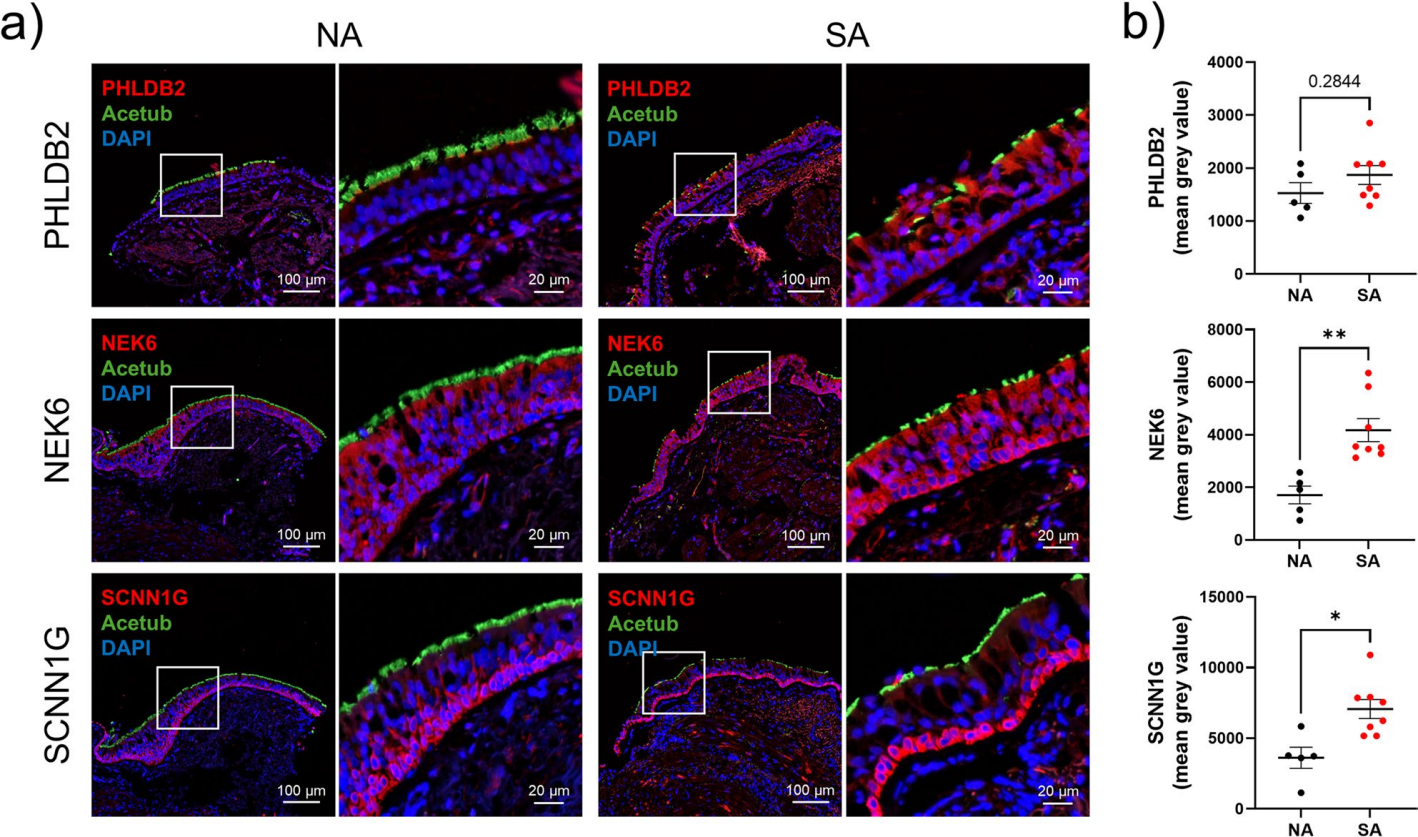
NEK6 and SCNN1G are altered in SA bronchial biopsies. **a** Representative micrographs showing bronchial epithelia of non-asthmatic (NA) and severe asthmatic (SA) patients immunostained for PHLDB2 (top panel, red), NEK6 (middle panel, red), SCNN1G (bottom panel, red), acetylated tubulin (Acetub, green) and cell nuclei (DAPI, blue). Magnification corresponding to the selected area is shown. **b** Dot plots with mean ± SEM representing bronchial PHLDB2 (top panel), NEK6 (middle panel) and SCNN1G (bottom panel) mean grey value of NA (*n* = 5, black) and SA (*n* = 8, red) patients. **p < 0.01; ***p < 0.001; NA vs. SA

The epithelial remodelling was assessed through secretory cell analysis (MUC5AC-, MUC5B-, and CC10-secreting cells), basal cell proportions (p63-positive cells), ciliated cell surface, and epithelium height (Fig. 2a). The abundances of MUC5AC-, MUC5B-, and CC10-secreting cells were not significantly different between the two groups. The surface of ciliated cells (37.7 ± 12.0% vs. 41 ± 19.3%, *p* = 0.6177) and epithelial height (60.3 ± 7.6 μm vs. 47.8 ± 7.3 μm, *p* = 0.2844) were not statistically different. Only, the proportion of basal cells was significantly lower in SA compared to NA (26.9%±2.4 vs. 37.1%±3.5, *p* = 0.0480), but it was not associated with the remodelling features (Fig. 2b). We investigated the localisation and expression of PHLDB2, NEK6, and SCNN1G in the epithelium of the bronchial biopsies. PHLDB2 was preferentially found at the base of cilia in NA patients, while it was identified in the cytoplasm of all AEC in SA patients. NEK6 was expressed in the entire epithelium in both NA and SA groups. SCNN1G was expressed in the basal side of the airway epithelium in NA patients, while it was more restricted to the cells completely attached to the basal lamina in SA patients (Fig. 3a). NEK6 and SCNN1G were significantly more abundant (respectively 4178 ± 437.5pmgv vs. 1713 ± 334pmgv, *p* = 0.0016; and 7073 ± 676.8pmgv vs. 3619 ± 745.9pmgv, *p* = 0.0109), while PHLDB2 analysis pointed towards an increase (1870 ± 178.6pmgv vs. 1529 ± 194.5pmgv, *p* = 0.2844) in SA compared to NA patients (Fig. 3b).

**Fig. 4:**
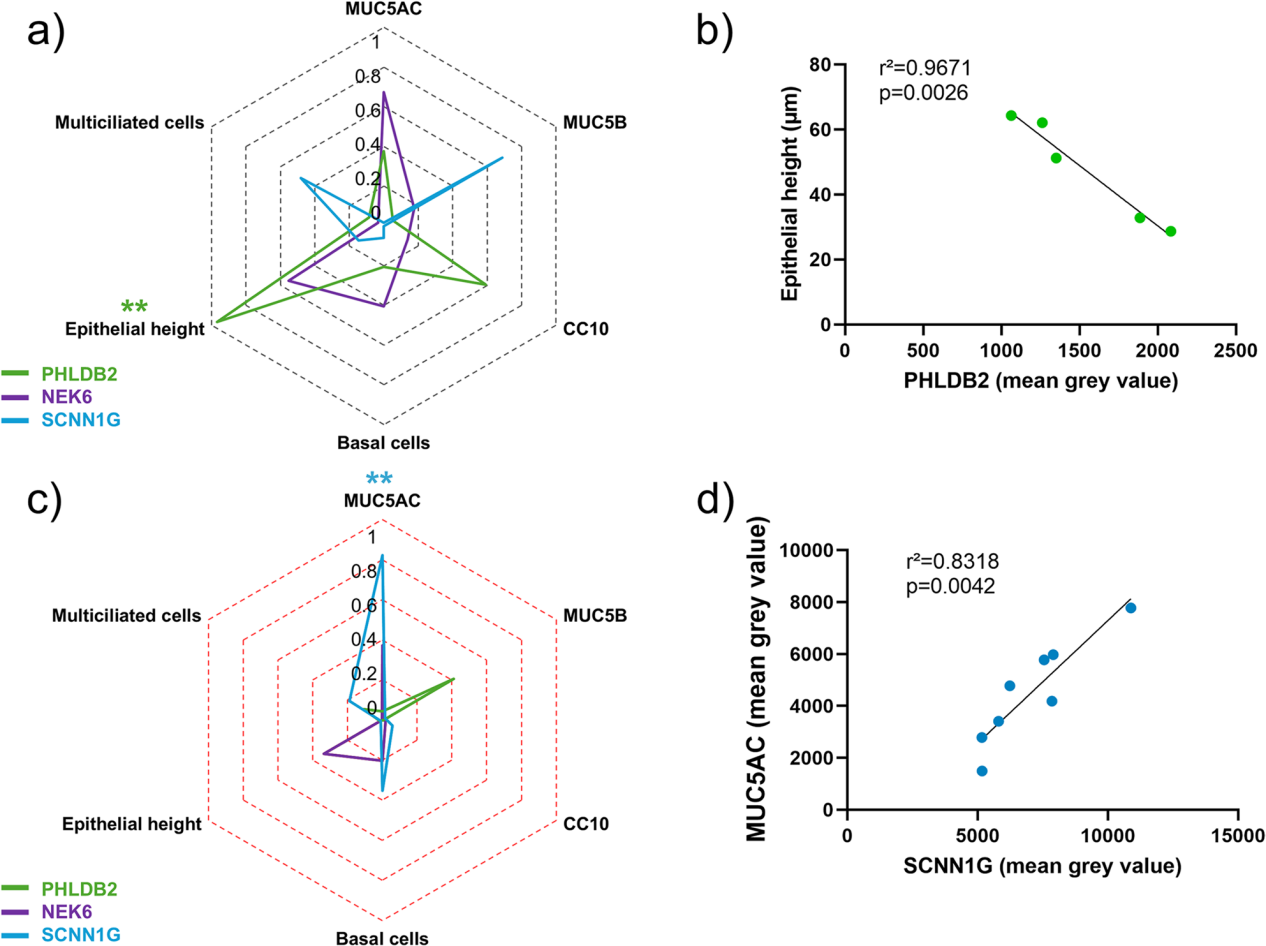
PHLDB2 and SCNN1G abundances are associated with epithelial remodelling features. **a** Radar chart displaying the square of the Pearson correlation *r* between the epithelial remodelling features and PHLDB2 (green line, *n* = 5), NEK6 (purple line, *n* = 5), SCNN1G (blue line, *n* = 5) bronchial expression in non-asthmatic (NA) patients. **b** Dot plot showing the linear regression for NA patients regarding PHLDB2 (green line, *n* = 5) bronchial expression and epithelial height. **p < 0.01; NA vs. SA. **c** Radar chart displaying the square of the Pearson correlation *r* between the epithelial remodelling features and PHLDB2 (green line, *n* = 8), NEK6 (purple line, *n* = 8), SCNN1G (blue line, *n* = 8) bronchial mean grey value in severe asthmatic (SA) patients. **d** Dot plot showing the linear regression for SA patients regarding SCNN1G (blue, *n* = 8) bronchial expression and the MUC5AC expression. **p < 0.01; NA vs. SA

**Fig. 5:**
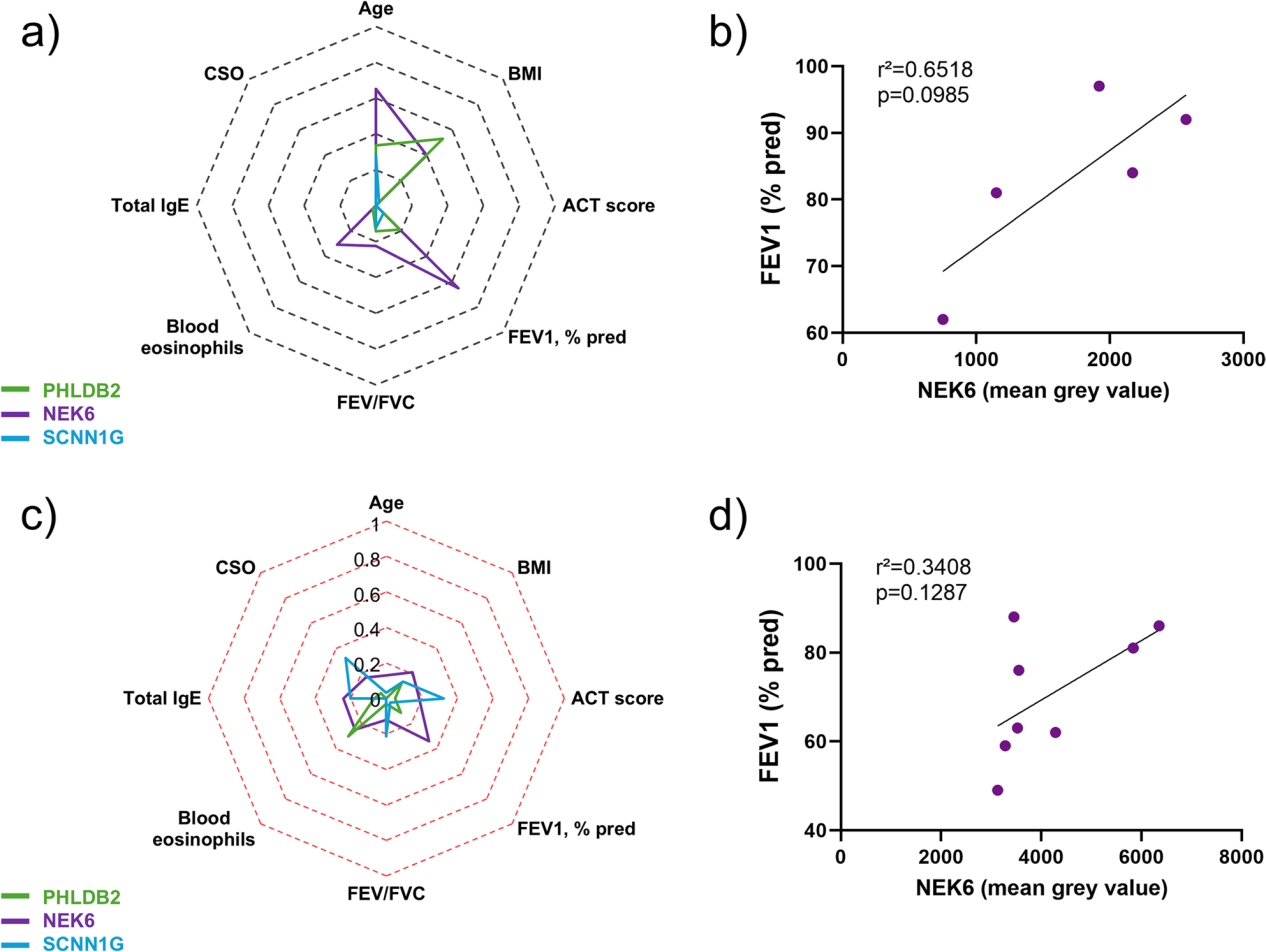
Bronchial expression of NEK6 tends to be associated with the respiratory function. **a** Radar chart displaying the square of the Pearson correlation *r* between the clinical data and PHLDB2 (green line, *n* = 5), NEK6 (purple line, *n* = 5), SCNN1G (blue line *n* = 5) bronchial expression in non-asthmatic (NA) patients. **b** Dot plot showing the linear regression for NA patients regarding NEK6 (purple line, *n* = 5) bronchial expression and the FEV_1_. **c** Radar chart displaying the square of the Pearson correlation *r* between the clinical data and PHLDB2 (green line, *n* = 8), NEK6 (purple line, *n* = 8), SCNN1G (blue line, *n* = 8) bronchial mean grey values in severe asthmatic (SA) patients. **d** Dot plot showing the linear regression for SA patients regarding NEK6 (purple, *n* = 8) bronchial expression and the FEV_1_

We investigated the correlations between PHLDB2, NEK6 or SCNN1G bronchial expression and epithelial remodelling features. In NA patients, the expression of PHLDB2 was significantly positively correlated to the bronchial epithelial height (r²=0.9671, *p* = 0.0026) (Fig. 4a and b). SCNN1G expression was significantly negatively associated with MUC5AC expression in SA patients (r²=0.8318, *p* = 0.0042) (Fig. 4c and d). There was no significant correlation between NEK6 bronchial expression and epithelial remodelling features in the NA and SA groups. We did not find any significant association between PHLDB2, NEK6 or SCNN1G bronchial expression and patients’ clinical characteristics (Fig. 5a and c). Interestingly, the bronchial expression of NEK6 tended to be positively correlated with predicted FEV_1_ in both groups (Fig. 5b and d).

### Evaluation of PHLDB2, NEK6 and SCNN1G during AEC differentiation

To further delve into the role of PHLDB2, NEK6, and SCNN1G in asthma, we explored their localisations and associations with remodelling and clinical features during AEC differentiation in NA (*n* = 6) and non-severe asthmatic patients (*n* = 6) (Supplementary Table S2). The two groups were comparable and showed no significant differences in terms of demographic or clinical features.

We investigated the epithelial remodelling features in human primary AEC from NA and asthmatic patients cultured in ALI for 14 days (differentiation in progress) with or without mimicking T2-high inflammation with IL-13 (Supplementary Fig. S3a). MUC5B-secreting cells were not detected in the cultures, while MUC5AC abundance was significantly higher in asthmatic AEC upon IL-13 stimulation (508.4 ± 91.7pmgv vs. 5117 ± 737.3pmgv, *p* = 0.0021) (Supplementary Fig. S3b). In contrast, this difference was not found between the control (CTL) and the IL-13-treated NA groups. There was no significant difference regarding the epithelial height in all groups. Interestingly, the ciliated surface and the proportion of basal cells were significantly altered with IL-13 treatment in both NA and asthmatic cultures. The ciliated surface was reduced 3.7-fold with IL-13 stimulation in NA (21.1 ± 12.3% vs. 77.4 ± 3.5%, *p* = 0.0313), and 6.9-fold in the asthmatic groups (10.1 ± 9.7% vs. 69.7 ± 4.7%, *p* = 0.0313). Similarly, the proportion of basal cells was increased with IL-13 stimulation in NA (34.1 ± 1.5% vs. 28.4 ± 1.5%, *p* = 0.0259) and asthmatic groups (37.0 ± 1.8% vs. 30.3 ± 1.1%, *p* = 0.0212). Interestingly, in the absence of IL-13, the proportion of p63-positive basal cells was associated with the detection of MUC5AC (r²=0.74, *p = 0.03). The TEER was significantly decreased with IL-13 stimulation in NA (377.8 ± 37.8Ω/cm² vs. 496.5 ± 47.3Ω/cm², *p* = 0.0094) and asthmatic cultures (367.8 ± 48.1Ω/cm² vs. 584.7 ± 27.3Ω/cm², *p* = 0.0118). There was no difference between CTL NA and CTL asthmatic cultures after 14 days of AEC differentiation for all the remodelling features (Supplementary Fig. S3). Altogether, after 14 days of differentiation in ALI, the epithelial remodelling induced by IL-13 was similar in NA and asthmatic cultures, including increased secretion of MUC5AC and proportion of basal cells, reduced ciliated surface and TEER.

Then, we investigated the localisation and expression of PHLDB2, NEK6, and SCNN1G after 14 days of ALI culture (Supplementary Fig. S4a). PHLDB2 expression was significantly decreased upon IL-13 stimulation in the NA group (1183 ± 105.7pmgv vs. 1737 ± 147.2pmgv, *p* = 0.0313) (Supplementary Fig. S4b) with a relative expression (CTL/IL-13) significantly decreased in the IL-13-treated NA group (0.7 ± 0.1, *p* = 0.0266 in NA vs. 0.9 ± 0.1, *p* = 0.4006 in the asthma group). There was no significant difference concerning NEK6 and SCNN1G relative expression between NA and asthma, or between the CTL and IL-13-treated groups (Supplementary Fig. S4b).

### Evaluation of PHLDB2, NEK6 and SCNN1G in fully differentiated epithelia

Finally, the epithelial remodelling features were analysed in human primary AEC from NA and asthmatic patients cultured in ALI for 28 days (fully differentiated epithelia) with or without IL-13 (Supplementary Fig. S5a). MUC5B-secreting cells were still not detected. There was no difference in epithelial height or the proportion of basal cells between all groups. In contrast, MUC5AC abundance was 8.3-fold significantly increased upon IL-13 stimulation in NA (8751 ± 692pmgv vs. 1051 ± 157.1pmgv; p = < 0.0001), and 4.8-fold in asthmatic cultures (7706 ± 1188pmgv vs. 1601 ± 244.5pmgv, *p* = 0.0013) (Supplementary Fig. S5b). There was a dramatic 3.4-fold decrease of the ciliated surface upon IL-13 stimulation in NA (27.7 ± 9.3% vs. 95.4 ± 0.7%, *p* = 0.001), and 3.7-fold in asthmatic cultures (25.9 ± 12.8% vs. 94.9 ± 0.8%, *p* = 0.0056). The TEER was significantly decreased with IL-13 stimulation in the NA (218.3 ± 24.7Ω/cm² vs. 367.7 ± 47.2Ω/cm², *p* = 0.0036) and asthmatic groups (305.7 ± 46.6Ω/cm² vs. 492.4 ± 78.5Ω/cm², *p* = 0.0056). Altogether, after 28 days of differentiation in ALI, the epithelial remodelling induced by IL-13 was similar in NA and asthmatic cultures, including increased secretion of MUC5AC, reduced ciliated surface, and increased TEER.

**Fig. 6:**
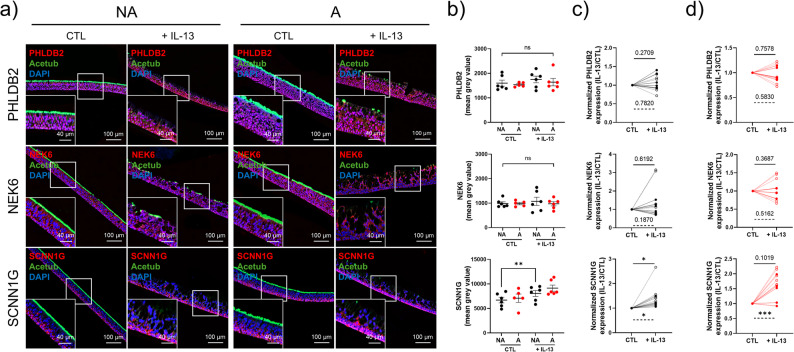
SCNN1G abundance is altered upon IL-13 treatment. **a** Representative micrographs showing epithelia of non-asthmatic (NA) and asthmatic (A) cultures, treated or not with IL-13 for 28 days in ALI-culture, and immunostained for PHLDB2 (top panel, red), NEK6 (middle panel, red), SCNN1G (bottom panel, red), acetylated tubulin (Acetub, green), and cell nuclei (DAPI, blue). Magnification corresponding to the selected area is shown. **b** Dot plots with mean ± SEM representing bronchial PHLDB2 (top panel), NEK6 (middle panel) and SCNN1G (bottom panel) mean grey values of NA (*n* = 6, black) and A (*n* = 6, red) patients with or without IL-13 stimulation for 28 days in ALI-culture. **p < 0.01; CTL vs. IL-13. **c** Before-after plots showing relative evolution of PHLDB2 (top panel), NEK6 (middle panel) and SCNN1G (bottom panel) expression of NA (*n* = 6, black) cultures after IL-13 stimulation for 28 days (solid line) and from 14 days to 28 days of culture (dashed line). *p < 0.05. **d** Before-after plots showing relative evolution of PHLDB2 (top panel), NEK6 (middle panel) and SCNN1G (bottom panel) expression of A (*n* = 5, red) cultures after IL-13 stimulation for 28 days (solid line) and from 14 days to 28 days of culture (dashed line)

The localisation and expression of PHLDB2, NEK6 and SCNN1G were investigated after 28 days of ALI culture (Fig. 6a). There was no significant difference regarding PHLDB2 and NEK6 localisation and relative abundances between the CTL and IL-13-treated cultures. SCNN1G expression was significantly increased upon IL-13 stimulation in NA cultures (8057 ± 603.1pmgv vs. 6704 ± 568.5, *p* = 0.0095) (Fig. 6b) with a relative expression (CTL/IL-13) significantly increased in IL-13-treated NA cultures (1.2 ± 0.1, *p* = 0.0188 in NA vs. 1.4 ± 0.2, *p* = 0.1019 in asthma). Furthermore, the PHLDB2 and NEK6 relative expressions from 14 days to 28 days of culture were not significant, while the SCNN1G relative expression was increased in both the NA and SA groups (respectively 1.6 ± 0.2, *p* = 0.0291; and 1.9 ± 0.1, *p* = 0.0003) (Fig. 6c and d).

**Fig. 7:**
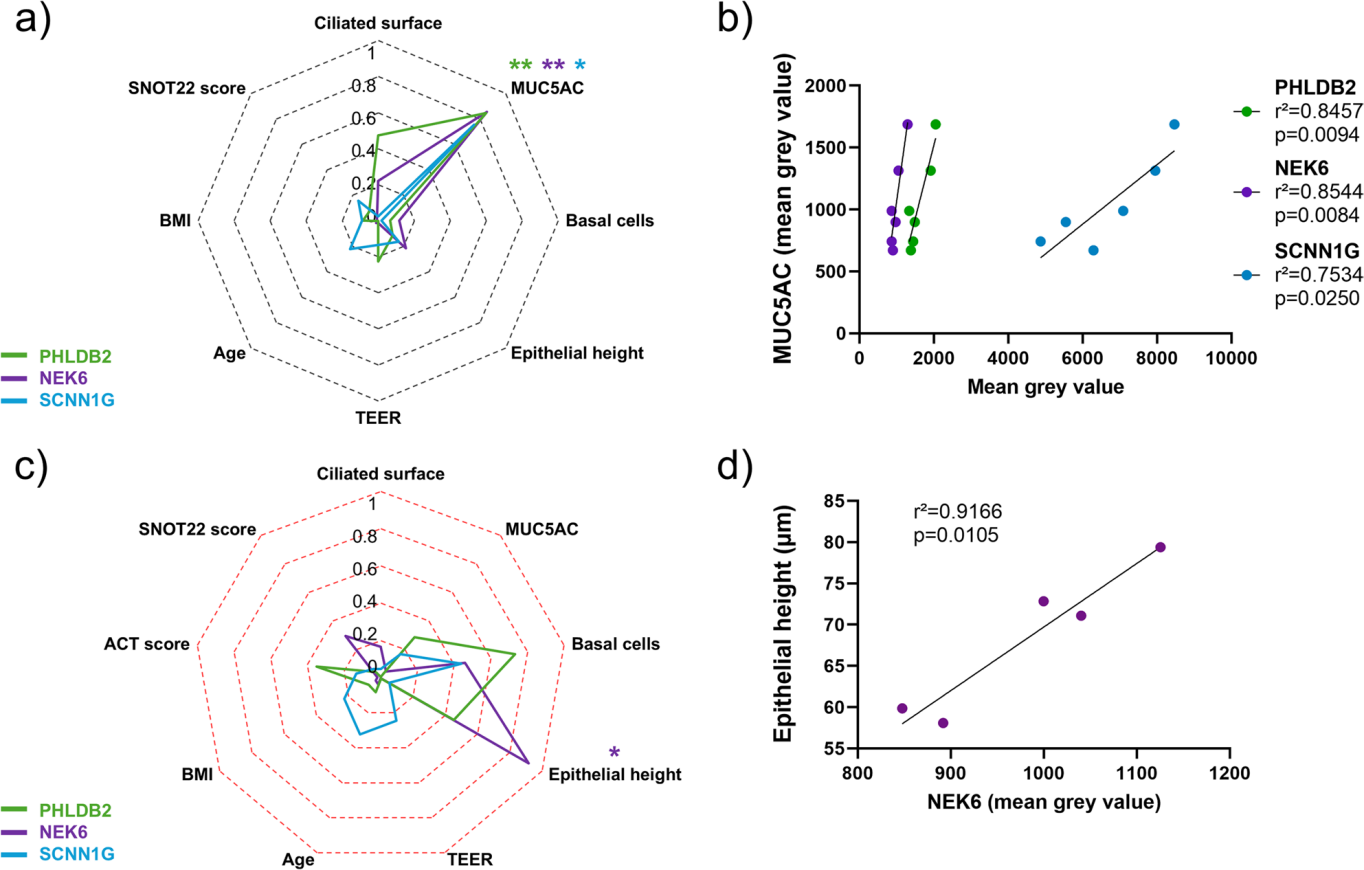
PHLDB2, NEK6 and SCNN1G are associated with epithelial remodelling in the absence of an inflammatory context. **a** Radar chart displaying the square of the Pearson correlation *r* between the epithelial remodelling features and clinical data, and PHLDB2 (green line), NEK6 (purple line), SCNN1G (blue line) expression in non-asthmatic (NA) cultures without IL-13 treatment. **b** Dot plot showing the linear regressions for NA patients regarding PHLDB2 (green, *n* = 6), NEK6 (purple, *n* = 6), and SCNN1G (blue, *n* = 6) expression and the MUC5AC expression. *p < 0.05; **p < 0.01. **c** Radar chart displaying the square of the Pearson correlation *r* between the clinical data and PHLDB2 (green line, *n* = 5), NEK6 (purple line, *n* = 5), and SCNN1G (blue line, *n* = 5) bronchial mean grey value in asthmatic cultures without IL-13 treatment. **d** Dot plot showing the linear regression for asthmatic cultures regarding NEK6 (purple, *n* = 5) bronchial expression and the epithelial height. *p < 0.05

**Fig. 8:**
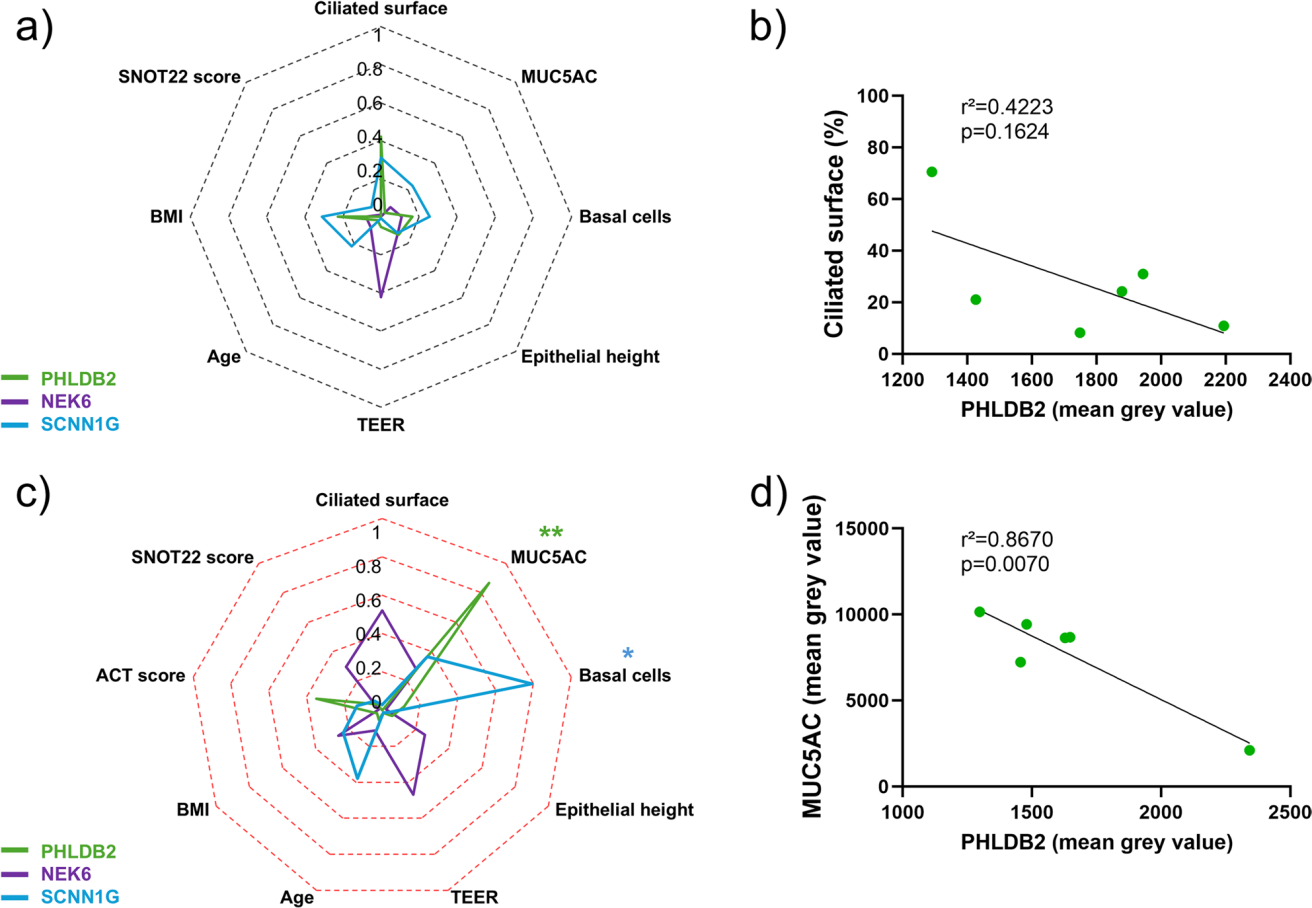
IL-13-treated asthmatic cultures highlight an association between PHLDB2 expression and MUC5AC. **a** Radar chart displaying the square of the Pearson correlation *r* between the epithelial remodelling features and clinical data, and PHLDB2 (green line, *n* = 6), NEK6 (purple line, *n* = 6), SCNN1G (blue line, *n* = 6) expression in non-asthmatic (NA) cultures treated with IL-13 for 28 days. **b** Dot plot showing the linear regression for NA cultures regarding PHLDB2 (green, *n* = 6) expression and the ciliated surface. **c** Radar chart displaying the square of the Pearson correlation *r* between the epithelial remodelling and clinical data, and PHLDB2 (green line, *n* = 5), NEK6 (purple line, *n* = 5), SCNN1G (blue line, *n* = 5), bronchial mean grey value in asthmatic cultures treated with IL-13 for 28 days. **d** Dot plot showing the linear regression for asthmatic cultures regarding PHLDB2 (green, *n* = 6) bronchial expression for the MUC5AC expression. *p < 0.05; **p < 0.01

We analysed the correlations between the abundances of PHLDB2, NEK6 or SCNN1G and the epithelial remodelling features or the clinical data of the patients after 28 days of ALI culture. Regarding the NA cultures, we highlighted significant positive associations between MUC5AC expression and PHLDB2 (r²=0.8457, *p* = 0.0094), NEK6 (r²=0.8544, *p* = 0.0084), and SCNN1G (r²=0.7435, *p* = 0.0250) expressions (Fig. 7a and b). In addition, an important positive correlation was observed between NEK6 expression and the epithelium height (r²=0.9166, *p* = 0.0105) in asthmatic cultures (Fig. 7c and d). There was no significant association in IL-13-treated NA cultures, but interestingly, the ciliated surface tended to be negatively correlated with PHLDB2 expression (r²=0.4223, *p* = 0.1624) (Fig. 8a and b). In contrast, the expression of PHLDB2 was negatively correlated with MUC5AC expression (r²=0.8670, *p* = 0.0070), and SCNN1G was positively correlated with the proportion of basal cells (r²=0.7915, *p* = 0.0176) in IL-13-treated asthmatic cultures (Fig. 8c and d).

## Discussion

We identified potential key molecular players potentially involved in bronchial epithelial remodelling, exploring cilia-associated dysregulations in patients with SA through 3 large biobanks of RNA sequencing. We investigated three cilia-associated molecular candidates that were commonly dysregulated in SA. Their expression was validated in both ex vivo and in vitro models, and these candidates were found to be associated with features of bronchial epithelial remodelling and clinical data.

Among the dysregulated cilia-associated genes, PHLDB2 (Pleckstrin homology-like domain family B member-2), NEK6 (NimA-related protein kinase 6), and SCNN1G (Epithelial sodium channel subunit gamma) emerged as key candidates potentially linked to airway epithelial remodelling in SA. PHLDB2 was initially characterised for its role in the post-synaptic apparatus, particularly in acetylcholine receptor clustering at the post-synaptic membrane [26]. However, its relevance in the pulmonary context has only recently emerged. Consistent with our results, PHLDB2 was found transcriptionally upregulated in the epithelium of patients with SA [27]. We also previously identified PHLDB2 among the 100 most dysregulated genes distinguishing SA with prominent cilia-associated defects from other SA, further supporting its association with ciliary dysfunction in asthma [18]. The second candidate, NEK6, is a serine/threonine kinase known to play a critical role in mitotic cell cycle progression [28, 29]. NEK6 was identified among the 20 most dysregulated genes in the peripheral blood of children with asthma, highlighting its potential involvement in systemic immune or inflammatory responses associated with this disease [30]. We previously identified NEK6 as a cilia- and ciliopathy-associated gene upregulated in the whole lung tissue of patients with COPD [16]. Finally, the SCNN1G gene encodes a subunit of the heterotrimeric epithelial sodium channel (ENaC), which plays a key role in regulating sodium balance and fluid homeostasis in lung epithelial cells [31]. Proper ENaC function is essential for maintaining the airway surface liquid that is crucial for mucus clearance [32]. Consistent with our findings, a previous study reported decreased SCNN1G transcript levels in epithelial brushings from asthmatic patients [33].

The analysis of protein expression and localisation of the three most dysregulated genes, PHLDB2, NEK6 and SCNN1G, in bronchial biopsies provided additional insights into their potential role in airway epithelial dysfunction. While PHLDB2 and NEK6 showed a protein upregulation consistent with their transcriptomic profiles, SCNN1G transcripts were downregulated while the protein levels were increased in SA. This discrepancy highlights the importance of post-transcriptional regulation in interpreting epithelial expressions. This divergence may also originate from the sampling methods. Bronchial brushings used for RNA sequencing usually collect superficial epithelial cells containing few basal cells, whereas protein expression was assessed in the whole bronchial epithelium obtained from biopsies. This is particularly relevant since SCNN1G was exclusively expressed on the basal side of the bronchial epithelium, with an expression more restricted to the cells completely attached to the basal lamina in SA.

Our findings suggest a differential involvement of PHLDB2, NEK6 and SCNN1G in bronchial epithelial remodelling between NA and SA patients. While overall epithelial remodelling features did not significantly differ between the two groups, the distinct correlations highlighted phenotype-specific molecular associations that may reflect divergent pathophysiological mechanisms. PHLDB2 expression correlated with epithelial height in NA. This protein has been previously implicated in cytoskeletal organisation and cell adhesion [34], suggesting that its involvement in epithelial structure may contribute to structural changes in asthma. This association with epithelial height might indicate a regulatory role of PHLDB2 in maintaining epithelial integrity. In the SA group, SCNN1G expression was associated with MUC5AC levels, pointing toward a link between ion transport regulation and mucous hypersecretion. The association of SCNN1G with MUC5AC, a mucin predominantly secreted by goblet cells, supports existing evidence linking ion transport imbalance with mucous phenotype in SA. This may reflect a dysregulation of epithelial secretory functions contributing to airway obstruction and disease severity.

The difference observed between protein expression profiles in bronchial biopsies and ALI-cultured AEC underscore the complexity of modelling the airway epithelial context. While bronchial biopsies represent a fully differentiated and contextually influenced airway epithelium (by the stromal and exogenous environments), ALI-cultures allow a controlled model to investigate epithelial differentiation over time, independent of the native tissue environment. In the absence of an inflammatory stimulus, NA and asthmatic ALI cultures were similar. The IL-13 treatment induced an important remodelling consisting of a large increase of MUC5AC-secreting cells and a dramatic decrease of the ciliated surface that was not exacerbated in asthmatic ALI cultures compared to NA. The lack of direct concordance between ex vivo and in vitro findings is not unexpected, as protein expression in vivo is shaped by a multitude of factors, including local inflammation, tissue microenvironment, mechanical stress, and cellular interactions, all of which are only partially represented in vitro. In addition, the heterogeneity in patient recruitment or sampling modalities (age, smoking history, severity, processing of the tissues, etc.), as well as the analysed outputs (type of remodelling features, analysis methods, etc.), may impact the results of a comparative study [35–38]. These findings emphasise the importance of integrating both in vitro and ex vivo approaches, as well as expanding the modulation of the AEC differentiation with additional inflammatory triggers or infections to better understand epithelial remodelling in asthma.

The significant upregulation of SCNN1G expression during chronic IL-13 stimulation (used to mimic key aspects of T2-high-driven remodelling observed in SA) and epithelial differentiation in ALI cultures further supports its potential involvement in chronic airway remodelling processes. Unlike PHLDB2 and NEK6, whose expression remained largely unchanged under IL-13 stimulation, SCNN1G showed both cytokine responsiveness and temporal regulation, with increased expression from day 14 to day 28 of cell differentiation. This suggests that SCNN1G may not only be a marker of disease severity, as observed in bronchial biopsies, but also be dynamically regulated during the epithelial differentiation under pro-inflammatory conditions.

The correlation analyses between the candidate molecules and epithelial remodelling features revealed distinct patterns depending on the disease status and IL-13 stimulation. In the NA group under control conditions, PHLDB2, NEK6, and SCNN1G were all positively associated with MUC5AC expression, suggesting a potential link with goblet cell differentiation and baseline mucin secretion in healthy epithelium. In contrast, in the asthmatic group without IL-13 stimulation, NEK6 expression was specifically associated with epithelial height, suggesting a role in the epithelial thickening characteristic of asthma pathology. Interestingly, IL-13 stimulation disrupted all correlations in the NA group, while in the asthmatic group, IL-13 exposure induced a shift in molecular associations: PHLDB2 became significantly correlated with MUC5AC, and SCNN1G expression was associated with the proportion of basal cells. These findings suggest that chronic T2-high inflammation may reprogram the functional relationships between cilia-associated genes and epithelial cell populations in asthma, potentially contributing to pathological remodelling. In a previous study, we identified a novel endotype of SA with a panel of cilia-associated genes that may contribute to epithelial remodelling in asthma through inflammatory modulation [18]. Based on these findings, investigating the correlation between the inflammatory profile and the expression of candidate molecules could help elucidate the biological processes through which they may influence airway epithelial remodelling.

Although we experimentally addressed the gene expression associations of PHLDB2, NEK6, and SCNN1G ex vivo and in vitro, the next important step would be the chronic modulation of these proteins throughout airway differentiation to complete their characterization. In the absence of validated pharmacological modulators, CRISPR-based techniques may provide a robust alternative to overcome the challenge of analysing gene-function impact in multiciliated cell differentiation, as well as in airway repair and remodelling [39, 40]. Considering our previous studies highlighting the role of primary cilia in cell differentiation under both healthy and COPD contexts [14, 17], investigating the expression and localisation of these candidates could provide novel insights into the role of cilia in asthma pathophysiology. The list of cilia-associated genes was not limited to motile cilia but also included primary cilia. Therefore, exploring the modulation of primary ciliogenesis represents a promising direction to assess the potential involvement of PHLDB2, NEK6, and SCNN1G in AEC differentiation and homeostasis. For instance, NEK6 was found necessary for cell cycle progression and dysregulated in COPD associated with cilia defects [16, 28, 29]. Because primary cilia are critical regulator of the cell cycle (G0 phase), NEK6 may represent a crucial target to understand the regulation of primary and motile cilia in asthma.

In conclusion, this study highlights the dysregulation of three cilia-associated genes, PHLDB2, NEK6, and SCNN1G, in the bronchial epithelium of patients with SA, both at transcript and protein levels. Their association with features of epithelial remodelling and altered differentiation under T2-high inflammatory conditions underscores their potential contribution to asthma pathogenesis. These findings provide a foundation for future investigations into their mechanistic roles in epithelial plasticity and ciliogenesis, and their potential as predictive biomarkers or therapeutic targets in SA.

## Supplementary Information

Supplementary Material 1.

## Data Availability

All data generated or analysed during this study are included in this published article and its supplementary information files. The datasets are publicly accessible via the data repository Gene Expression Omnibus (GEO): GSE76226, GSE63142 and GSE158752.

## References

[1] Yuan L, Tao J, Wang J, She W, Zou Y, Li R, et al. Global, regional, National burden of asthma from 1990 to 2021, with projections of incidence to 2050: a systematic analysis of the global burden of disease study 2021. EClinicalMedicine. 2025;80:103051. 10.1016/j.eclinm.2024.103051. 39867965 10.1016/j.eclinm.2024.103051 PMC11764843

[2] Dharmage SC, Perret JL, Custovic A. Epidemiology of asthma in children and adults. Front Pediatr. 2019;7:246. 10.3389/fped.2019.00246. 31275909 10.3389/fped.2019.00246 PMC6591438

[3] Kuruvilla ME, Lee FE-H, Lee GB. Understanding asthma Phenotypes, Endotypes, and mechanisms of disease. Clin Rev Allergy Immunol. 2019;56:219–33. 10.1007/s12016-018-8712-1. 30206782 10.1007/s12016-018-8712-1 PMC6411459

[4] Kaur R, Chupp G. Phenotypes and endotypes of adult asthma: moving toward precision medicine. J Allergy Clin Immunol. 2019;144:1–12. 10.1016/j.jaci.2019.05.031. 31277742 10.1016/j.jaci.2019.05.031

[5] Hekking P-PW, Wener RR, Amelink M, Zwinderman AH, Bouvy ML, Bel EH. The prevalence of severe refractory asthma. J Allergy Clin Immunol. 2015;135:896–902. 10.1016/j.jaci.2014.08.042. 25441637 10.1016/j.jaci.2014.08.042

[6] Heijink IH, Kuchibhotla VNS, Roffel MP, Maes T, Knight DA, Sayers I, et al. Epithelial cell dysfunction, a major driver of asthma development. Allergy. 2020;75:1902–17. 10.1111/all.14421. 32460363 10.1111/all.14421 PMC7496351

[7] Yang SK, Kubo S, Black CS, Peri K, Dai D, Legal T, et al. Effect of α-tubulin acetylation on the doublet microtubule structure. eLife. 2024;12:RP92219. 10.7554/eLife.92219. 38598282 10.7554/eLife.92219 PMC11006419

[8] Zhang Q, Hu J, Ling K. Molecular views of Arf-like small GTPases in cilia and ciliopathies. Exp Cell Res. 2013;319:2316–22. 10.1016/j.yexcr.2013.03.024. 23548655 10.1016/j.yexcr.2013.03.024 PMC3742637

[9] Thomas B, Rutman A, Hirst RA, Haldar P, Wardlaw AJ, Bankart J, et al. Ciliary dysfunction and ultrastructural abnormalities are features of severe asthma. J Allergy Clin Immunol. 2010;126:722–e7292. 10.1016/j.jaci.2010.05.046. 20673980 10.1016/j.jaci.2010.05.046

[10] Corcoran TE, Huber AS, Hill SL, Locke LW, Weber L, Muthukrishnan A, et al. Mucociliary clearance differs in mild asthma by levels of type 2 inflammation. Chest. 2021;160:1604–13. 10.1016/j.chest.2021.05.013. 34029561 10.1016/j.chest.2021.05.013 PMC8628176

[11] Jackson ND, Everman JL, Chioccioli M, Feriani L, Goldfarbmuren KC, Sajuthi SP, et al. Single-Cell and population transcriptomics reveal Pan-epithelial remodeling in type 2-High asthma. Cell Rep. 2020;32:107872. 10.1016/j.celrep.2020.107872. 32640237 10.1016/j.celrep.2020.107872 PMC8046336

[12] Gerovac BJ, Fregien NL. IL-13 inhibits multicilin expression and ciliogenesis via Janus Kinase/Signal transducer and activator of transcription independently of Notch cleavage. Am J Respir Cell Mol Biol. 2016;54:554–61. 10.1165/rcmb.2015-0227OC. 26414872 10.1165/rcmb.2015-0227OC

[13] Kim H, Lee Y-S, Kim S-M, Jang S, Choi H, Lee J-W, et al. RNA demethylation by FTO stabilizes the FOXJ1 mRNA for proper motile ciliogenesis. Dev Cell. 2021;56:1118–e11306. 10.1016/j.devcel.2021.03.006. 33761320 10.1016/j.devcel.2021.03.006

[14] Belgacemi R, Diabasana Z, Hoarau A, Dubernard X, Mérol J-C, Ruaux C, et al. Primary ciliogenesis is a crucial step for multiciliated cell determinism in the respiratory epithelium. J Cell Mol Med. 2021;25:7575–9. 10.1111/jcmm.16729. 34170075 10.1111/jcmm.16729 PMC8335676

[15] Petit LMG, Belgacemi R, Ancel J, Saber Cherif L, Polette M, Perotin J-M, et al. Airway ciliated cells in adult lung homeostasis and COPD. Eur Respir Rev Off J Eur Respir Soc. 2023;32:230106. 10.1183/16000617.0106-2023. 10.1183/16000617.0106-2023 PMC10698550 38056888

[16] Perotin J-M, Polette M, Deslée G, Dormoy V. CiliOPD: a ciliopathy-associated COPD endotype. Respir Res. 2021;22:74. 10.1186/s12931-021-01665-4. 33639936 10.1186/s12931-021-01665-4 PMC7912836

[17] Perotin J-M, Coraux C, Lagonotte E, Birembaut P, Delepine G, Polette M, et al. Alteration of primary cilia in COPD. Eur Respir J. 2018;52:1800122. 10.1183/13993003.00122-2018. 29678947 10.1183/13993003.00122-2018

[18] Devilliers MA, Brisebarre A, Petit LMG, Polette M, Deslée G, Djukanović R, et al. Airway epithelial cell cilia transcriptomic dysregulation is associated with the inflammatory phenotype in asthma. Allergy. 2024;79:1982–8. 10.1111/all.16063. 38372076 10.1111/all.16063

[19] Perotin J-M, Schofield JPR, Wilson SJ, Ward J, Brandsma J, Strazzeri F, et al. Epithelial dysregulation in obese severe asthmatics with gastro-oesophageal reflux. Eur Respir J. 2019;53:1900453. 10.1183/13993003.00453-2019. 31023846 10.1183/13993003.00453-2019 PMC7610816

[20] Camiolo M, Gauthier M, Kaminski N, Ray A, Wenzel SE. Expression of SARS-CoV-2 receptor ACE2 and coincident host response signature varies by asthma inflammatory phenotype. J Allergy Clin Immunol. 2020;146:315–e3247. 10.1016/j.jaci.2020.05.051. 32531372 10.1016/j.jaci.2020.05.051 PMC7283064

[21] Camiolo MJ, Zhou X, Wei Q, Trejo Bittar HE, Kaminski N, Ray A, et al. Machine learning implicates the IL-18 signaling axis in severe asthma. JCI Insight. 2021;6:e149945. 10.1172/jci.insight.149945. 34591794 10.1172/jci.insight.149945 PMC8663569

[22] Modena BD, Tedrow JR, Milosevic J, Bleecker ER, Meyers DA, Wu W, et al. Gene expression in relation to exhaled nitric oxide identifies novel asthma phenotypes with unique biomolecular pathways. Am J Respir Crit Care Med. 2014;190:1363–72. 10.1164/rccm.201406-1099OC. 25338189 10.1164/rccm.201406-1099OC PMC4294630

[23] Vieira Braga FA, Kar G, Berg M, Carpaij OA, Polanski K, Simon LM, et al. A cellular census of human lungs identifies novel cell States in health and in asthma. Nat Med. 2019;25:1153–63. 10.1038/s41591-019-0468-5. 31209336 10.1038/s41591-019-0468-5

[24] Ladjemi MZ, Di Candia L, Heddebaut N, Techoueyres C, Airaud E, Soussan D, et al. Clinical and histopathologic predictors of therapeutic response to bronchial thermoplasty in severe refractory asthma. J Allergy Clin Immunol. 2021;148:1227–e12356. 10.1016/j.jaci.2020.12.642. 33453288 10.1016/j.jaci.2020.12.642

[25] Ce L, Gd A, Mp E, Ra K. Arl13b regulates ciliogenesis and the dynamic localization of Shh signaling proteins. Mol Biol Cell. 2011;22. 10.1091/mbc.E10-12-0994. 10.1091/mbc.E10-12-0994 PMC3226485 21976698

[26] Kishi M, Kummer TT, Eglen SJ, Sanes JR. LL5beta: a regulator of postsynaptic differentiation identified in a screen for synaptically enriched transcripts at the neuromuscular junction. J Cell Biol. 2005;169:355–66. 10.1083/jcb.200411012. 15851520 10.1083/jcb.200411012 PMC2171857

[27] de Jong E, Bosco A. Dissecting asthma transcriptomics: does site matter? Am J Respir Cell Mol Biol. 2018;58:144–6. 10.1165/rcmb.2017-0360ED. 29388830 10.1165/rcmb.2017-0360ED

[28] Yin M-J, Shao L, Voehringer D, Smeal T, Jallal B. The serine/threonine kinase Nek6 is required for cell cycle progression through mitosis. J Biol Chem. 2003;278:52454–60. 10.1074/jbc.M308080200. 14563848 10.1074/jbc.M308080200

[29] Belham C, Comb MJ, Avruch J. Identification of the NIMA family kinases NEK6/7 as regulators of the p70 ribosomal S6 kinase. Curr Biol CB. 2001;11:1155–67. 10.1016/s0960-9822(01)00369-4. 11516946 10.1016/s0960-9822(01)00369-4

[30] Wang C, Li H, Cao L, Wang G. Identification of differentially expressed genes associated with asthma in children based on the bioanalysis of the regulatory network. Mol Med Rep. 2018;18:2153–63. 10.3892/mmr.2018.9205. 29956778 10.3892/mmr.2018.9205 PMC6072229

[31] Zhao C, Crosby J, Lv T, Bai D, Monia BP, Guo S. Antisense oligonucleotide targeting of mRNAs encoding ENaC subunits α, β, and γ improves cystic fibrosis-like disease in mice. J Cyst Fibros Off J Eur Cyst Fibros Soc. 2019;18:334–41. 10.1016/j.jcf.2018.07.006. 10.1016/j.jcf.2018.07.006 30100257

[32] Wang W, Ji H-L. Epithelial sodium and chloride channels and asthma. Chin Med J (Engl). 2015;128:2242–9. 10.4103/0366-6999.162494. 26265620 10.4103/0366-6999.162494 PMC4717984

[33] Woodruff PG, Boushey HA, Dolganov GM, Barker CS, Yang YH, Donnelly S, et al. Genome-wide profiling identifies epithelial cell genes associated with asthma and with treatment response to corticosteroids. Proc Natl Acad Sci U S A. 2007;104:15858–63. 10.1073/pnas.0707413104. 17898169 10.1073/pnas.0707413104 PMC2000427

[34] Hotta A, Kawakatsu T, Nakatani T, Sato T, Matsui C, Sukezane T, et al. Laminin-based cell adhesion anchors microtubule plus ends to the epithelial cell basal cortex through LL5alpha/beta. J Cell Biol. 2010;189:901–17. 10.1083/jcb.200910095. 20513769 10.1083/jcb.200910095 PMC2878951

[35] Gras D, Bourdin A, Vachier I, de Senneville L, Bonnans C, Chanez P. An ex vivo model of severe asthma using reconstituted human bronchial epithelium. J Allergy Clin Immunol. 2012;129:1259–e12661. 10.1016/j.jaci.2012.01.073. 22409990 10.1016/j.jaci.2012.01.073

[36] Ordoñez CL, Khashayar R, Wong HH, Ferrando R, Wu R, Hyde DM, et al. Mild and moderate asthma is associated with airway goblet cell hyperplasia and abnormalities in mucin gene expression. Am J Respir Crit Care Med. 2001;163:517–23. 10.1164/ajrccm.163.2.2004039. 11179133 10.1164/ajrccm.163.2.2004039

[37] Varricchi G, Brightling CE, Grainge C, Lambrecht BN, Chanez P. Airway remodelling in asthma and the epithelium: on the edge of a new era. Eur Respir J. 2024;63:2301619. 10.1183/13993003.01619-2023. 38609094 10.1183/13993003.01619-2023 PMC11024394

[38] Bourdin A, Neveu D, Vachier I, Paganin F, Godard P, Chanez P. Specificity of basement membrane thickening in severe asthma. J Allergy Clin Immunol. 2007;119:1367–74. 10.1016/j.jaci.2007.01.055. 17481707 10.1016/j.jaci.2007.01.055

[39] Zaragosi L-E, Gouleau A, Delin M, Lebrigand K, Arguel M-J, Girard-Riboulleau C, et al. Combination of CRISPR-Cas9-RNP and Single-Cell RNAseq to identify cell State-Specific FOXJ1 functions in the human airway epithelium. Methods Mol Biol Clifton NJ. 2024;2725:1–25. 10.1007/978-1-0716-3507-0_1. 10.1007/978-1-0716-3507-0_1 37856015

[40] Rapiteanu R, Karagyozova T, Zimmermann N, Singh K, Wayne G, Martufi M, et al. Highly efficient genome editing in primary human bronchial epithelial cells differentiated at air-liquid interface. Eur Respir J. 2020;55:1900950. 10.1183/13993003.00950-2019. 32060058 10.1183/13993003.00950-2019

