# Supplementary material for: Identification and validation of cilia-associated molecular candidates deregulated in severe asthma
Source: Respir Res. 2026 Feb 9;27:128. doi: 10.1186/s12931-026-03548-y (PMC12983791; doi:10.1186/s12931-026-03548-y)
Supplement: Supplementary file 1 — Supplementary Material 1. [file 12931_2026_3548_MOESM1_ESM.docx]

**Identification and validation of cilia-associated molecular candidates deregulated in severe asthma**

Maëva A. Devilliers, Lynda Saber Cherif, Audrey Brisebarre, Ruby Chouquet, Ludivine Bralet, Julien Ancel, Alexandre Vivien, Emilie Luczka-Majérus, Arnaud Bonnomet, Nathalie Lalun, Camille Taillé, Xavier Dubernard, Jean-Claude Mérol, Christophe Ruaux, Myriam Polette, Gaëtan Deslée, Jeanne-Marie Perotin, Valérian Dormoy

**Supplementary material**

Supplementary figures:

Figure S1: Multi-subject single-cell (MuSiC) deconvolution

Figure S2: Validation of the immunofluorescence candidate detection

Figure S3: Airway remodelling is enhanced upon IL-13 treatment at ALI-14

Figure S4: PHLDB2 abundance is altered upon IL-13 treatment at ALI-14

Figure S5: Airway remodelling is enhanced upon IL-13 treatment at ALI-28

Supplementary tables:

Table S1**:** List of primary antibodies used for FFPE immunostainings

Table S2: Clinical characteristics of the patients included for FFPE immunostainings on airway epithelial cells cultured in ALI-culture

**Supplementary figures**

Figure S1. Multi-subject single-cell (MuSiC) deconvolution. (a) Barplots showing estimated cell-type proportions in three bulk RNA-seq datasets from HC (top panels) and SA (bottom panels) patients. Each bar represents a bulk sample, and colors indicate the relative proportion of each cell type. (b) Barplots showing estimated airway epithelial cell proportions in bulk RNA-seq datasets from HC (top panel) and SA (bottom panel) patients. Each bar represents a bulk sample, and colors indicate the relative proportion of each cell type.


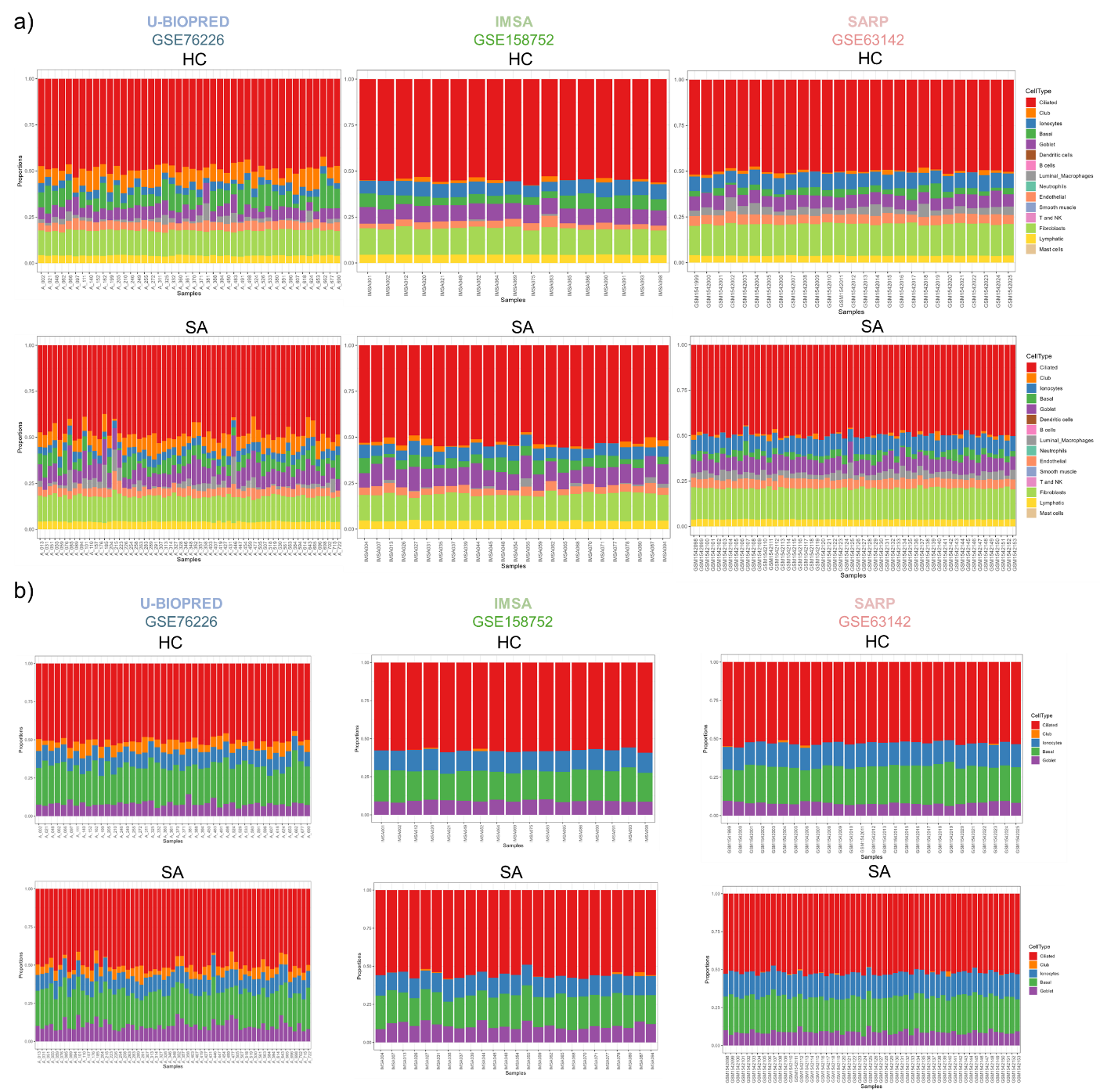


Figure S2: Validation of the immunofluorescence candidate detection. (a) Representative micrographs showing bronchial biospies immunostained with the indicated primary antibodies (red, right panel) or without primary antibodies as a negative control (left panel). DAPI (blue) marks nuclei. (b) Representative micrographs showing IAL cultures immunostained with the indicated primary antibodies (red, right panel) or without primary antibodies as a negative control (left panel). DAPI (blue) marks nuclei. The absence of signal in the negative control confirms the specificity of the primary antibodies used.


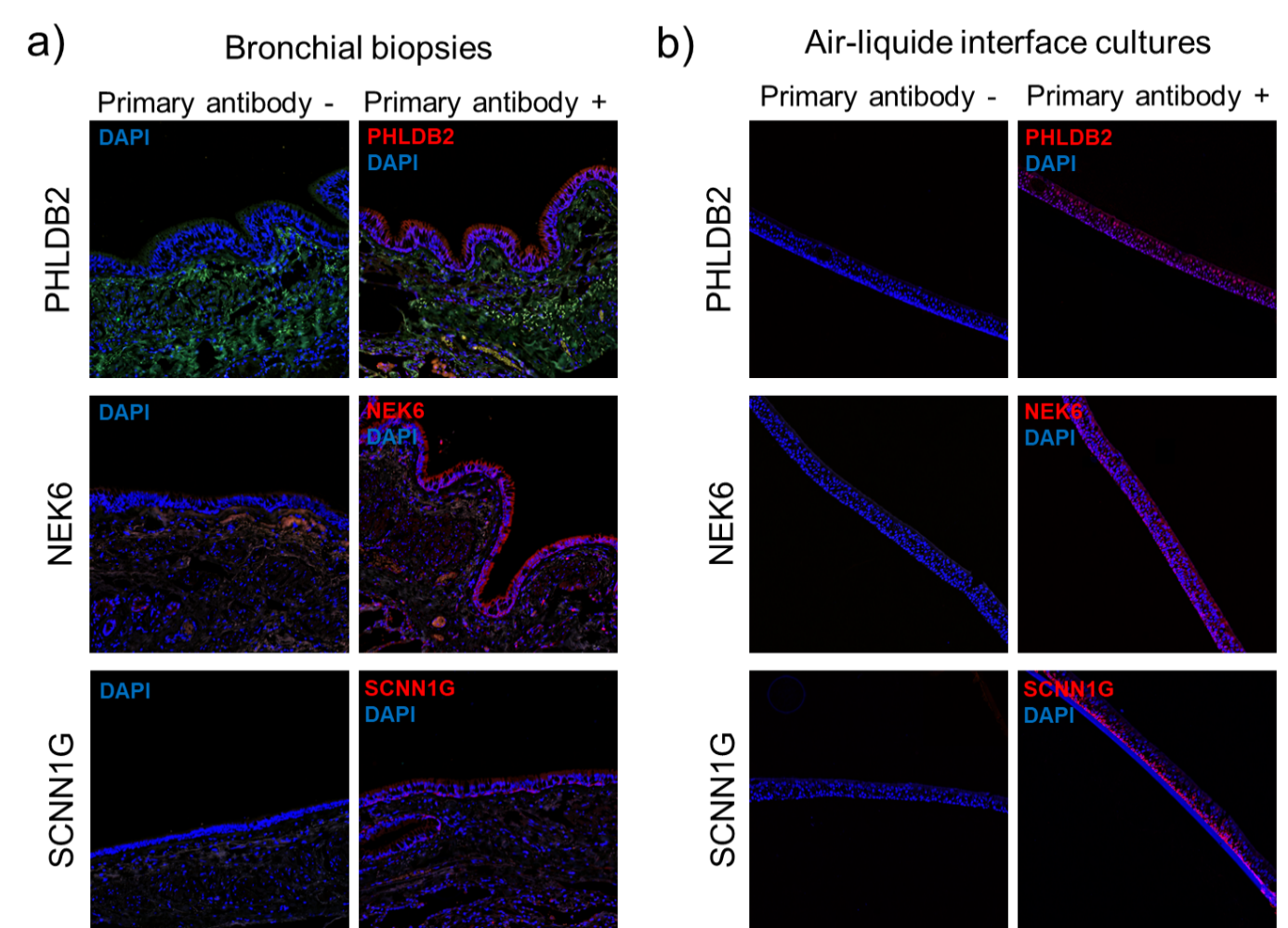


Figure S3: Airway remodelling is enhanced upon IL-13 treatment at ALI-14. (a) Representative micrographs showing epithelia of non-asthmatic (NA) and asthmatic (A) ALI cultures, treated or not with IL-13 for 14 days, and immunostained for epithelial remodelling features MUC5AC/MUC5B (top panel), ARL13B/p63 (middle panel) and cell nuclei (DAPI, blue). Magnification corresponding to the selected area is shown. (b) Dot plots with mean ± SEM representing MUC5AC expression, epithelial height, proportion of basal cells and ciliated surface, and TEER of NA (n=6, black) and A (n=6, red) cultures treated or not with IL-13 for 14 days. *p<0.05; **p<0.01; CTL vs IL-13.


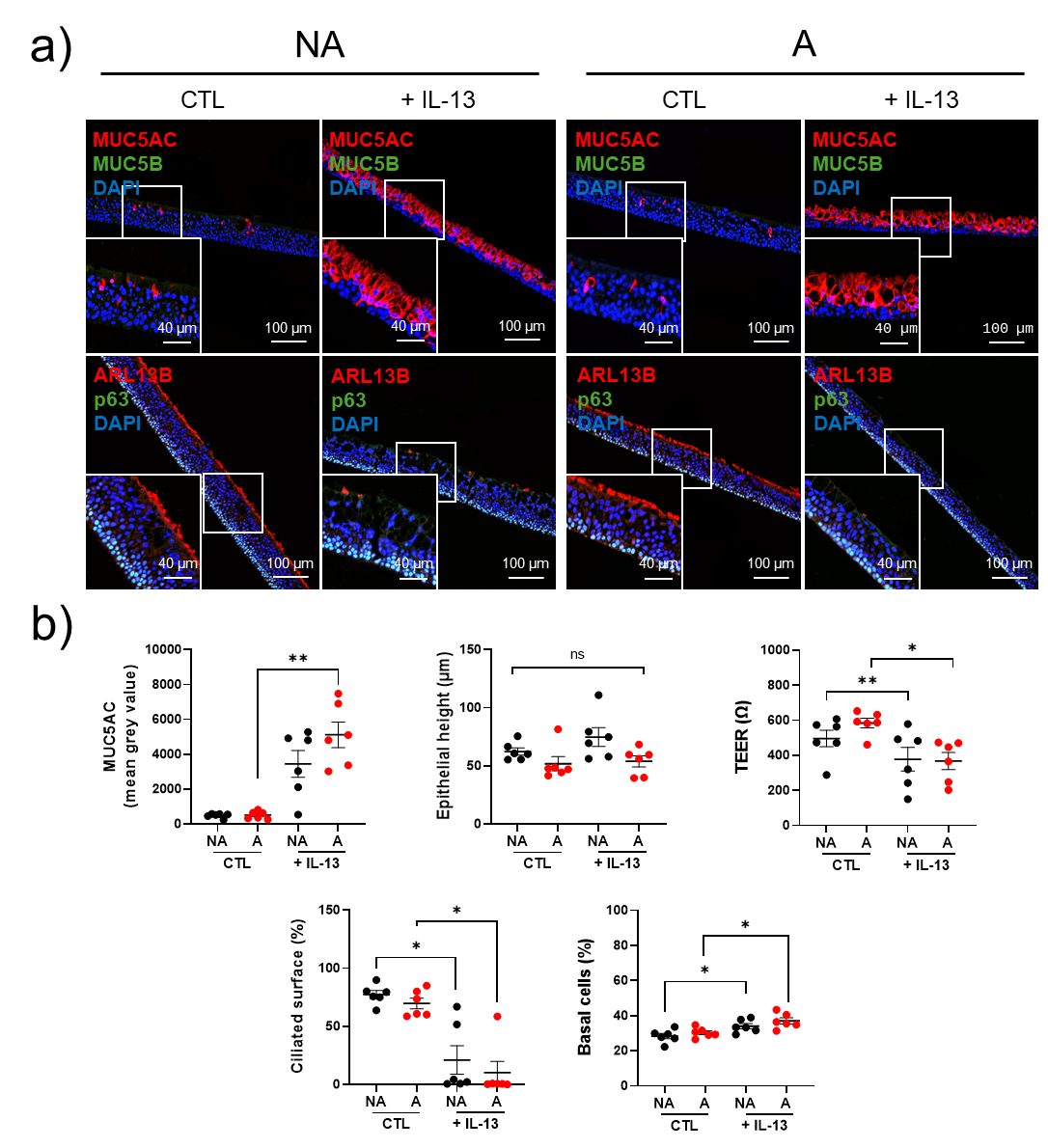


Figure S4: PHLDB2 abundance is altered upon IL-13 treatment at ALI-14. (a) Representative micrographs showing epithelia of non-asthmatic (NA) and asthmatic (A) ALI cultures, treated or not with IL-13 for 14 days, and immunostained for PHLDB2 (top panel, red), NEK6 (middle panel, red), SCNN1G (bottom panel, red), acetylated tubulin (Acetub, green) and cell nuclei (DAPI, blue). Magnification corresponding to the selected area is shown. (b) Dot plots with mean ± SEM representing PHLDB2 (top panel), NEK6 (middle panel), SCNN1G (bottom panel) expression of NA (n=6, black) and A (n=6, red) cultures treated or not with IL-13 for 14 days. *p<0.05; **p<0.01. (c) Before-after plots showing relative evolution of PHLDB2 (top panel), NEK6 (middle panel) and SCNN1G (bottom panel) expression of NA (n=6, black) cultures after IL-13 stimulation for 14 days. (d) Before-after plots showing relative evolution of PHLDB2 (top panel), NEK6 (middle panel) and SCNN1G (bottom panel) expression of A (n=5, red) cultures after IL-13 stimulation for 14 days in ALI-culture. *p<0.05.


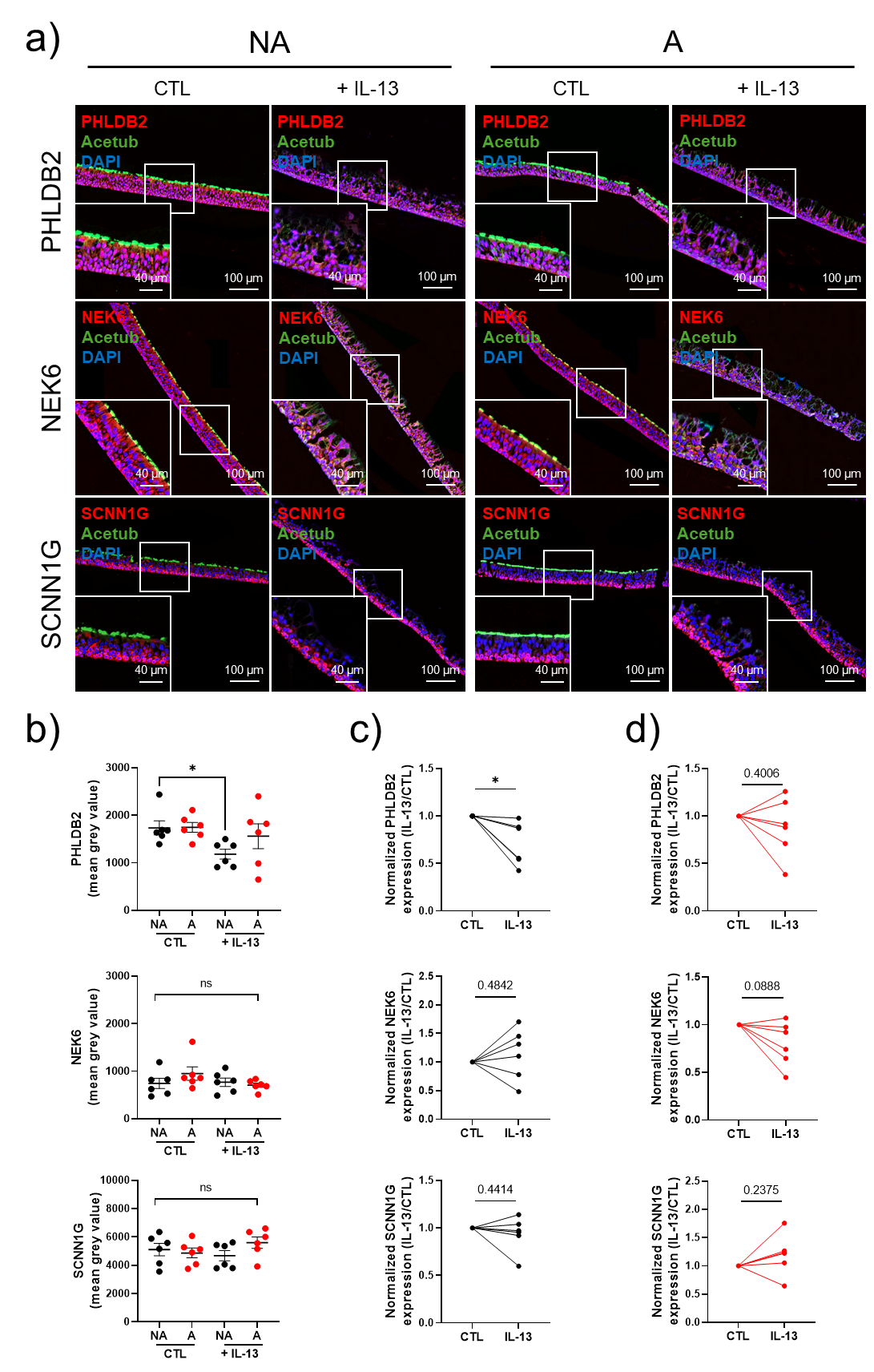


Figure S5: Airway remodelling is enhanced upon IL-13 treatment at ALI-28. (a) Representative micrographs showing epithelia of non-asthmatic (NA) and asthmatic (A) cultures, treated or not with IL-13 for 28 days, and immunostained for epithelial remodelling features MUC5AC/MUC5B (top panel), ARL13B/p63 (middle panel) and cell nuclei (DAPI, blue). Magnification corresponding to the selected area is shown. (b) Dot plots with mean ± SEM representing MUC5AC expression, epithelial height, proportion of basal cells and ciliated surface, and TEER of NA (n=6, black) and A (n=5, red) cultures treated or not with IL-13 for 28 days. *p<0.05; **p<0.01; CTL vs IL-13.


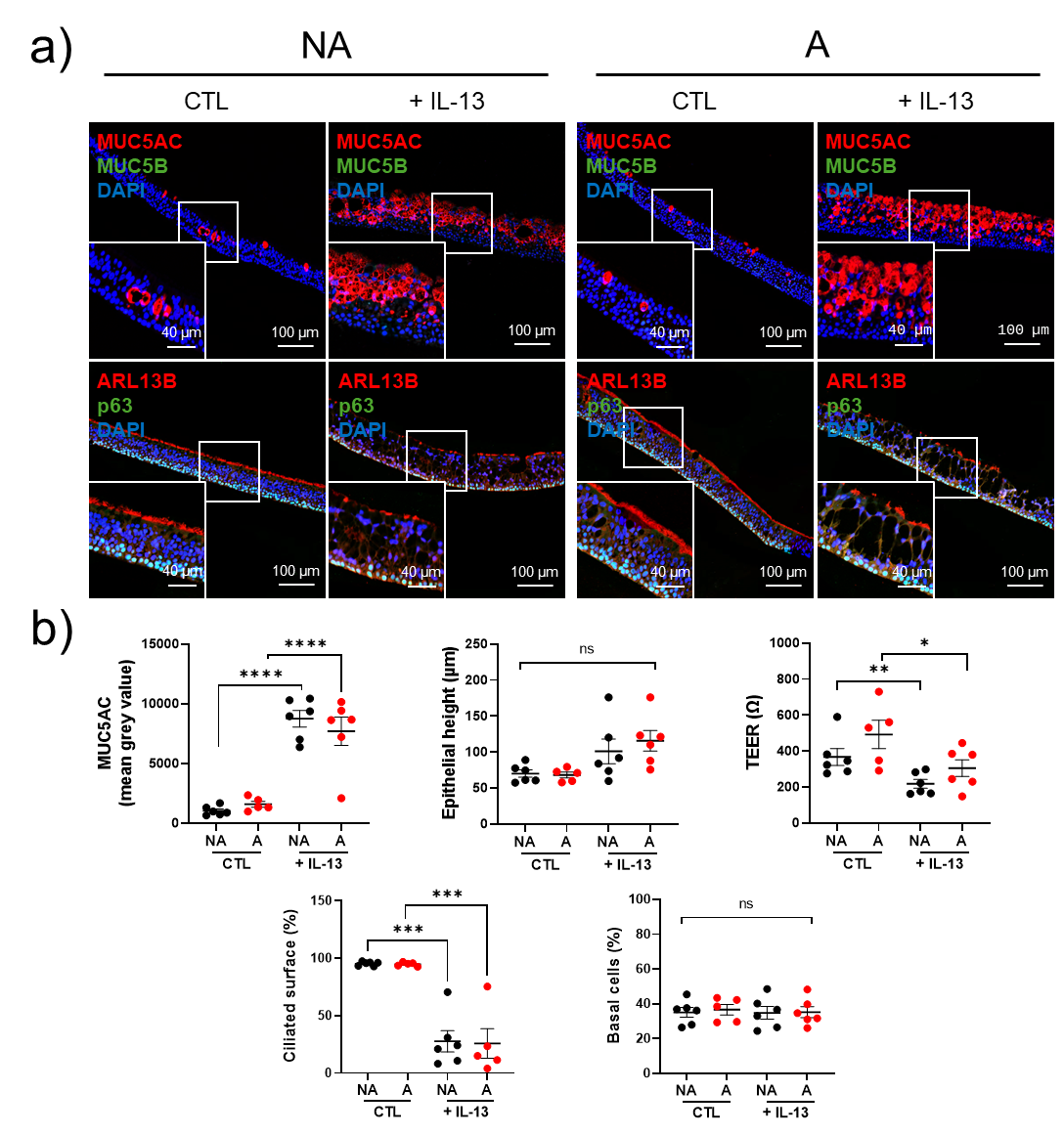


**Supplementary tables**

Table S1**:** List of primary antibodies used for FFPE immunostainings.

| **Antibodies** | **Species** | **References** | **Companies** | **Concentrations** | **RRID** |
| --- | --- | --- | --- | --- | --- |
| Acetylated tubulin | Mouse | T6793 | Sigma-Aldrich | IF – 1:1000 | AB_477585 |
| Arl13b | Rabbit | 17711-1-AP | Proteintech | IF – 1:100 | AB_2060867 |
| CC10 | Rabbit | 10490-1-AP | Proteintech | IF – 1:100 | AB_2183285 |
| Muc5ac | Mouse | MAB2011 | Millipore | IF – 1:100 | AB_2146983 |
| Muc5b | Rabbit | E-AB-15988 | Elabscience | IF – 1:200 | AB_3107123 |
| NEK6 | Rabbit | HPA056828 | Sigma-Aldrich | IF – 1:100 | AB_2683250 |
| P63 | Goat | AF1916 | R&D Systems | IF – 1:100 | AB_2207174 |
| PHLDB2 | Rabbit | NBP2-38238 | Novus Biologicals | IF – 1:100 | AB_3297699 |
| SCNN1G | Rabbit | 13943-1-AP | Proteintech | IF – 1:500 | AB_2184510 |

Table S2: Clinical characteristics of the patients included for FFPE immunostainings on airway epithelial cells cultured in ALI-culture.

| Clusters | A | NA | p value |
| --- | --- | --- | --- |
| n | 6 | 6 |  |
| *Demography and clinical characteristics* | | | |
| Age at inclusion (yrs) | 64.2 ± 11.4 | 53.4 ± 13.3 | 0.099 |
| Sex -F/M | 1/5 | 2/4 | >0.999 |
| BMI^a^ (kg/m²) | 26.82 ± 8.2 | 26.6 ± 7.2 | 0.964 |
| ACT^b^ score | 22.5 [18-25] | ND |  |
| SNOT22^c^ score | 38.0 [29-56] | 47.3 [2-90] | 0.523 |
| *Smoking history* |  |  |  |
| Never smokers | 6 (100) | 4 (66.7) | 0.455 |
| Current smokers | 0 (0) | 2 (33.3) | 0.455 |
| Pack-years | ND | 14.0 [10-18] |  |

^a^ BMI: Body mass index; ^b^ ACT: Asthma control test; ^c^ SNOT22: Sino-Nasal Outcome Test 22. ND: Not determined.
